# Clinical Review and Hospital Reports

**Published:** 1835-04-01

**Authors:** 


### Clinical Review.

Glasgow Royal Infirmary.

Report on Tumors. By Dr. Macfarlane*.

We have at various times extracted many important facts from the volume of Clinical Reports before us. That volume being strictly a collection of cases is of permanent utility and character; at all events, it has served and will serve us again for a granary, from which to extract sound and wholesome nutriment.

The subject of tumors has occupied the attention and exercised the ingenuity of surgeons of eminence and talent. Their aim has been to nosologize the varieties of morbid growths, and to classify and distinguish their separate forms. Were these forms determinate, and were they *always* separate, the task would still be difficult, and the attempt might fail. But, unhappily, various tumors are convertible into each other, and what commenced as a simple enlargement of some natural structure, may, under circumstances with which we are imperfectly, if at all, acquainted, become converted into the most formidable malignant alteration. When we add to this vital difficulty the consideration that the external distinctive marks of tumors are absolutely vague, that their differences of structure are imperfectly appreciable by dissection, and that, little as we know, the powers of description are unable accurately to convey even that, we think we may offer a reasonable solution of the causes why so little real progress has been made in this department of our science. We are rather surprised at what has, than at what has not been done, at the definiteness of our knowledge, not its looseness. How much has been effected since the days of Mr. Pott and Mr. Hey in determining the history of scirrhus and of fungus hæmatodes—how gratifying is our power of saving, in consequence of our advance in diagnosis, many breasts and many testicles that would formerly, we mean twenty years ago, have been sacrificed. None can bound the progress of human knowledge, none can say, "Thus far and no farther wilt thou go." The sciences are so dovetailed and entangled, that the progress of one drags the other forward. If morbid anatomy would seem to have exerted almost all its strength in accomplishing diagnosis and in circling treatment, we cannot affirm, and we will not suppose that chemistry and botany have exhausted theirs.

Sixty-one pages of Dr. Macfarlane's volume are dedicated to the report on Tumors. The magnitude of the space is not disproportioned to that of the subject. The hospital surgeon, constantly in the habit of witnessing such cases, may peruse with indifference reports of this description. But hospital surgeons are a trifling minority in the profession, and the members who unfortunately are debarred from the advantages which those gentlemen possess, must find in the pages of journals or of books, the best substitute for them which they can. The philosophical principle of the greatest good to the greatest number, is as applicable in the world of medicine as of politics.

Dr. Macfarlane adopts the classification of tumors originally proposed by Mr. Abernethy. It is not good, but it is the best. Our readers, then, must understand by adipose sarcoma, pancreatic sarcoma, and so forth, tumors possessed of the characters described under such designations, by the eminent surgeon to whom we have alluded. For the sake of convenience of description, our author reports on tumors of the head and neck, tumors of the mamma, and, finally, tumors of the abdomen.

I. Tumors of the Head and Neck.

Adipose tumors of the scalp are un-

* Clinical Reports of the Surgical Practice of the Glasgow Royal Infirmary. By J. Macfarlane, M.D.

frequent. Dr. Macfarlane relates two cases, in one of which the disease was confined to the subcutaneous structure, whilst in the other it was situated beneath the occipito-frontalis. We shall abbreviate the already abbreviated details.

CASE 1. "W. J., æt. fifty-four, had a large, prominent, well-defined, and doughy tumour, about the size of a small orange, situated over the centre of the left parietal bone. Its origin was attributed to a blow he had received on the part about three years before, and since that time it had been slowly increasing. It was freely moveable over the subjacent parts, but firmly adherent to the integuments, which retained their natural colour. Its surface was traversed by several enlarged veins. It was broader and more expanded at the apex than at the base;* and it only gave him pain when compressed by the hat, or when otherwise subjected to external irritation."

The tumor appearing to be adipose sarcoma was removed from the subcutaneous cellular tissue to which it was confined. It was enveloped in a fine cyst, and in the centre the adeps was considerably condensed. On the third day erysipelas supervened, superficial sloughing of the wound ensued, but the patient ultimately recovered.

CASE 2. *Adipose Sarcoma beneath the Occipito-frontalis.*

"J.G., æt, forty-seven, had had a soft, flat, ill-defined tumour, growing over the centre of the occipital bone, for about five years, when he applied at the Infirmary to have it extirpated, in August, 1831, and for which he had previously used a variety of local applications without benefit. On proceeding to remove it with the knife, I found it covered by, and intimately adhering to, the occipito-frontalis muscle, which was much thickened. The wound healed without difficulty, and no untoward occurrence took place. On dissecting the tumor, it was found to be composed of adipose matter, contained in a distinct cyst, and much flattened in shape by the resistance to its developement, produce by the tendinous expansion under which it was situated."

We need only allude to two observations of our author's. He, then, is satisfied, and, probably, he is correct, that where sutures are employed for retaining the edges of a scalp wound in contact, erysipelas is more frequent than when ordinary dressings are resorted to. The second observation has reference to the difference observed in the tumor in the two preceding cases. In the first, where little compression was exercised, the growth was pendulous and large; in the last it was broad, and flat, and ill-defined, in consequence of the pressure of the occipito-frontalis. It was on the observation of such facts as these that the treatment of cancer by compression was founded, a treatment which was not found to succeed.

Dr. M. relates an interesting case of encysted tumor of the scalp; interesting, because it illustrates the fact that a morbid growth, which is commonly innocent, may, under peculiar circumstances, assume a malignant character. Before we make any further remark we will lay the case before our readers.

CASE 3. *Encysted Tumor of the Scalp Fungating.*

A. M'D., æt. sixty-five, entered the Infirmary on the 18th June, 1826, to have a tumor removed from his head. It was situated over the centre of the left parietal bone, to which it adhered intimately, and was about the size of a pigeon's egg. It was deeply ulcerated; the edges were thickened and everted; the surface had an irregular cauliflower appearance; the discharge was ichorous; and the pain acute and lancinating. This tumor had existed for eleven years in an innocent state, similar to other two of the common encysted kind, on the opposite side of the head; when, after a bruise, it became painful, inflamed, and ulcerated. It was extirpated. Erysipelas succeeded,

* We do not exactly perceive how a thing can be broader at the apex than the base. This looks like a Hibernicism.—*Eds.*

and the wound was long in cicatrizing, but the patient has had no return of the disease.

Sir Benjamin Brodie is in the habit of mentioning, in his Clinical Lectures, more than one case of this description. Dr. Macfarlane remarks that he has witnessed two other cases of the kind. In one of them, amputation of the penis for cancer had been resorted to a few months before the tumor on the head assumed the appearance of malignity. This patient died from the propagation of the disease to the inguinal glands.

It has been frequently observed by judicious surgeons, that malignancy and non-malignancy are terms which should not be too positively employed as distinctive characters of tumors. There are various grades of both conditions, and, perhaps, the very extremes are convertible. With respect to malignant tumors, this is certain—that some possess in a much more eminent degree than others the power of contaminating the general system. Fungus hæmatodes is more virulent in this respect than scirrhus, and from scirrhus to some simple chronic enlargements of glandular structure, the shades are numerous and the distinctions faint. The encysted tumors which fungate, as in Dr. Macfarlane's case, appear to influence the system but slightly, perhaps they may not sensibly injure it at all. Yet they do not seem susceptible of a natural cure, and the knife is required more for their removal, than for the preservation of the constitution.

Tumors in the neck are frequent, and their removal by an operation is often required. Those operations are delicate and dangerous from the situation and complexity of the great cervical nerves and vessels, and the depth and extent of the connexions of the morbid growth. Those tumors which arise, as some do, from the external surface of the platysma muscle are, of course, not deep, though they may be extensive; they are, therefore, extirpated with comparative facility. But when the tumor springs from beneath the fasciæ, a knowledge of anatomy would lead us to expect what experience serves to confirm, that the operation is proportionately difficult. The first case we shall cite is one of chronic abscess in the neck.

Case 4. *Chronic Abscess in the Neck.*
C. M'P. æt. 20, admitted 12th April, 1832, having been sent by a surgeon, from the Island of Mull, to have a tumor extracted from her neck. It was seated in the left side, having the sterno-mastoid muscle for its anterior, and the trapezius for its posterior boundary. It dipped under the middle of the clavicle, and extended to within an inch of the mastoid process of the temporal bone. It was covered by sound integuments, and had a firm, resisting feel, except a small spot in the centre, where obscure fluctuation was discovered. When first observed, a year and half before, it was about the size of a pea. It increased slowly, but never was the seat of acute pain. Her general health was good.

A consultation produced, as usual, several opinions. Dr. Macfarlane, however, punctured the tumor, and evacuated seven ounces of healthy-looking pus. The sac gradually filled by granulations, and a cure ensued.

"When," says our author, "an encysted tumor forms in the subcutaneous cellular texture, the fluid nature of its contents can be easily ascertained; but when it is situated below the fascia, its progress externally will be considerably impeded, and the sense of fluctuation rendered much more obscure. Even in this latter situation, however, the nature of the tumour may be correctly ascertained by careful and deliberate examination, except when the cyst is greatly thickened. But it sometimes happens that the cyst, although thin, is so completely distended with the fluid, as to give the tumour a hard, tense feel. When a tumor, so distended, is situated in the abdominal parietes, I have seen expert surgeons foiled in detecting fluctuation, because they could not fix it against any hard resisting body, so that the requisite degree of pressure might be applied. It is still more difficult to ascertain, before it has been punctured whether the sac contains pus or serum. Every surgeon of common observation must have seen chronic abscesses in which little or no pain was present, and where months elapsed before the tumors attained to any great size. In such cases it is hardly possible, by external examination or by accurate attention to the history of the disease, to know whether the tumor is filled with pus or serum. Nor is the distinction, in general, of much practical importance."

Dr. Macfarlane does not allude to an important means of diagnosis in all tumors, an experimentum crucis in all but very deep ones. We allude to the operation of puncturing the tumor with a grooved needle. The fluid, if there be any, lodges in the groove, and betrays the contents of the tumor. The surgeon is ignorant or confident who hesitates to avail himself of this useful mode of exploration.

In the last number of this Journal we analysed a paper by Dr. O'Beirne, of Dublin, on encysted tumors of the neck containing water. That paper is highly deserving of attention, and its perusal may be associated with the cases we are now relating.

Acute abscess beneath the fascia of the neck is unnoticed by our author. Yet it is a serious and not very uncommon affection, and its seriousness is perhaps increased by the mistakes often made as to its nature. The tumor spreads laterally, rather than advances towards the surface, the fluctuation is obscure, the redness of the surface not considerable, and generally there is œdema of the subcutaneous tissue. The general symptoms are at first those of inflammatory, afterwards those of typhoid fever. If incisions are not made the integuments slough extensively, so do the deep cellular tissue and the fascia, and very formidable the mischief is. We have seen the integuments of nearly one side of the neck destroyed, and the lower jaw exposed, and killed by mismanaged abscess of this description. The treatment is obvious, and should be decisive—early and deep openings, assisted by all powerful antiphlogistic local treatment.

We proceed to another subject.

CASE 5. *Encysted Tumor over the Parotid Gland—Extirpation—Salivary Fistula.*

J. S., æt. 24, admitted 10th December, 1826. First observed, about eighteen months before, without any evident cause, a small elastic tumor, below the lobe of the right ear, and between the angle of the jaw and the mastoid process of the temporal bone. It was globular, had an elastic fluctuating feel, was larger than a hen's egg, and free of pain, even when roughly handled. It was covered by healthy integuments, and appeared to dip rather deeply, and to impede the free movement of the lower jaw.

The tumor was evidently encysted, its contents fluid, and Dr. Macfarlane determined to dissect it out. It was found firmly bound down by the fascia. During the dissection the sac was accidentally opened by a hook, when a considerable quantity of limpid fluid was discharged. The relaxed cyst, which was found adhering intimately to the outer edge of the masseter muscle, to the angle of the inferior maxilla, to the interior edge of the sterno-mastoid muscle, to the parotid gland, and to the cartilage of the external ear, was then separated, leaving a deep cavity behind the angle of the jaw. On its being dissected from the parotid, a small portion of the capsule, was removed, and the granular texture of the gland exposed.

Erysipelas came on about the sixth or seventh day, but, on the 21st of January, the patient was dismissed with the wound healed to a mere point, from which no saliva was discharged. Three weeks, however, after leaving the infirmary, she became subject, during mastication, to profuse discharges of watery fluid, through a small opening in the centre of the cicatrix, which had never healed. By applying the nitras argenti freely and frequently to the fistulous opening, and to the surface of the parotid gland from which the saliva flowed, and by the use of firm and continued pressure, for some weeks,—by means of graduated compresses and a bandage,—a cure was accomplished.

"We are told by Burns, that the inferior lobe of the parotid gland may become sacculated, so as to give rise to a tumor behind the angle of the jaw, formed by an accumulation of saliva. When this happens, I presume we are entitled to expect that there shall be a direct communication between the gland and the cyst; for upon no other principle can we account for the gradual increase of saliva, which must take place as the tumor enlarges. Should this explanation hold good, then it follows that in the above case the tumor was a common encysted, and not a salivary one, attached to, but not incorporated with the parotid gland; because, on examining the cyst, which was removed entire, there was no opening found on its posterior surface by which fluid could be conveyed to it from the parotid. This opinion is not invalidated by the subsequent occurrence of fistula, which owed its origin to the accidental injury of the gland during the removal of the cyst."

Two cases of tumor in the neck are next detailed. One had the characters of medullary, the other of tuberculated sarcoma.

CASE 6. *Medullary Sarcoma (?) in the left side of the Neck—Operation.*

To abridge the features of the case or the description of the operation would only be to deprive both of their value. We therefore subjoin them in the narrator's words.

"M. S., aged forty-six, was admitted on the 5th of February, 1827, and the following particulars of her case entered in the journal:—

'There is situated over the angle of the jaw, on the left side, a tumor, considerably larger than the fist, which extends from the edge of the sterno-mastoid muscle, as forward as the chin. It has an irregular shape; is distinctly lobulated on the surface, to which the integuments are firmly adhering; projects considerably, and is somewhat flattened on its summit. It has a firm, resisting, but in some parts a slightly elastic feel; is closely attached to the parts beneath; and its surface is traversed by the external jugular, and by several other venous branches of considerable size. When examined from the mouth, it appears to be firmly fixed to the lower jaw, below the alveolar processes; but it can only be felt indistinctly, by pressing with the finger under the tongue. It measures in circumference, at its base, twelve inches, and at its apex seven inches. Its diameter from above, downwards, and from before, backwards, is five inches; and it projects two inches beyond the jaw-bone, and about three inches beyond the edge of the mastoid muscle.

The tumor, when first observed, nine months ago, was about the size of a small nut: it was seated between the sterno-mastoid and the angle of the jaw, and was hard, moveable, and free of pain. It increased rapidly—became the seat of obtuse pain; and she says that, during the last fourteen days that portion of it which stretches to the angle of the mouth has formed. It impedes deglutition and mastication, and she thinks has affected her health.'

"On the 10th of February, the tumor was extirpated, but with greater difficulty than I expected. It adhered very intimately to the common integuments, and the fascia covering it was much thickened. I began to detach it at the posterior part, where it passed deeply under the angle of the jaw towards the base of the cranium, and proceeded forwards, dissecting it from the side of the face, from the inferior maxilla, and from the neck over the larynx and trachea. It adhered firmly to the lower jaw for about two-and-a-half inches; and here the bone was denuded of periosteum, rough, and had a cribriform appearance. Two of the submaxillary glands, which were enlarged, were also removed; and the several parts with which the tumor was connected were distinctly seen. It had extended back as far as the styloid process of the temporal bone; and besides the sterno-mastoid, digastric masseter and buccinator muscles, which were more or less exposed, the sheath of the vessels was laid bare, through which the pulsations of the carotid were visible. Five arteries were tied; and as there was little chance of procuring adhesion, the hollow under the angle of the jaw was filled with lint. Two stitches were inserted, and the parts supported by straps, compress, and bandage. The hæmorrhage which occurred was chiefly venous, and took place from the superficial veins, and from a large vein attached to the anterior part of the tumour. The external jugular escaped being injured.

On examining the tumour after its removal, it presented a soft, greyish-coloured texture, not unlike carcinoma, but without the fibrous bands or stony hardness peculiar to this morbid growth. In the centre, there was a cyst about the size of a walnut, which contained a soft, greyish-coloured matter, about the consistence of cream. This was mixed with clots of blood; and there were three similar cysts in other parts of the tumour, corresponding to the nodules observed externally previous to the operation."

On the next day, there were difficulty of swallowing and dyspnœa, apparently dependent on irritation of the bottom of the wound. These symptoms continued for four days, after which nothing untoward occurred, and she was speedily dismissed cured.

Our author observes, and we think with justice, that the tumor observed a closer similarity to medullary sarcoma, than to any other morbid growth. We need scarcely tell our readers, and we certainly need not hint to Dr. Macfarlane, that the healing of the wound, and dismissal of the patient, are seldom in such cases synonymous with ultimate recovery. Too many patients have been said to have been cured by operations which ultimately failed, operations, we mean, performed for the extirpation of malignant tumors. Those bloody beacons, like the false lights of wreckers, have blazed but to betray, and the surgeon and the patient have often been lured on by their lying lustre, to perform and to submit to barbarous repetitions of equally unsuccessful butchery. Our author does not tell, and we will not guess, what the termination of the last case has been. But if the disease was really what it seemed to be—medullary sarcoma, it will sin agreeably against our experience, if that termination has not been disastrous.

Case 7.—*Tubercular Sarcoma in Left Side of Neck.*

Mr. M'G. æt. 45, admitted Sept. 27, 1831. There was situated behind the left angle of the jaw, a firm, irregular, and partially circumscribed tumor, about the size of a hen's egg, which was somewhat flattened, and admitted of very limited motion. It extended from the mastoid process over the ramus of the jaw, and appeared to pass deeply behind the angle of that bone. It pressed up the lobe of the ear, and extended along the cheek, fully half-an-inch beyond the usual situation of the parotid duct. This tumor, when observed for the first time, three years before, was about the size of a walnut; but it was only within the last four months that it began to increase perceptibly, and to be accompanied with pain.

"On slightly depressing the head towards the left shoulder, and grasping the tumour firmly, it admitted of such a degree of motion, even in the limited and confined space in which it was situated, as led me to believe that it was capable of being extirpated. Accordingly, on the 30th, I succeeded in removing it, after a cautious dissection. Its posterior attachments were so firm and intimate, that they had to be divided with the knife, which was done close to the tumour. It was found covered by, and intimately adhering to, the inferior lobe of the parotid gland, which was also removed. The parotid duct did not present itself to view, but every precaution was taken to avoid it. Only two arteries required the ligature; and one of these was the occipital, about an inch of which was intimately adhering to, and removed along with the tumour. The edges of the wound were retained in apposition by three points of suture, over which a thick compress and double-headed roller were applied."

Erysipelas followed, but subsided under antiphlogistic treatment. On the 20th of October she was dismissed cured.

Examination of the tumor disclosed the following characters :—It had an irregular shape, a tuberose surface, and was surrounded by a dense fibrous covering. A section of it displayed an assemblage of small yellowish-coloured tubercles, about the size of mustard-seeds, apparently united by cellular texture. There were a few white fibrous bands in the centre; and near the circumference, where the tumor was attached to the parotid, there was a small portion, about the size of a sixpence, where the texture was more distinctly granular, and resembled that of one of the salivary glands.

Dr. Macfarlane remarks, with perfect propriety, on the excessive difficulty of classifying these tumors, even after their structure has been exposed by sections. The fact is, that the arrangement recommended by Mr. Abernethy, though better, perhaps, than any other, is a bad one, wanting, as it does, the most essential requisite for an accurate classification, distinctions which are readily appreciable. This is principally due to the convertibility of morbid structures—to the want of any real barrier between many which are separated by art. The tubercular, pancreatic, and medullary sarcoma run into each other, and surgeons can seldom be found to agree on the nature of the tumor prior to an operation; sometimes, indeed, not after its structure has been exposed by the knife. Perhaps the difficulties attending an accurate diagnosis are insuperable; perhaps they may be conquered by chemistry, or the minute anatomy of structure. A confession of our present ignorance is indispensable.

A remark or two on tubercular sarcoma, and we pass to tumors of the breast. Dr. Macfarlane observes, with respect to it—

"Mr. Abernethy considers tuberculated sarcoma to be a very malignant disease; but I have had several opportunities of observing that this is not always the case. I have seen more than one instance in which such tumours have remained for years in a perfectly quiescent state, without exciting either local pain or constitutional disturbance. I recollect, in particular, of examining, when in Strathaven, in the summer of 1827, a young, healthy, athletic man, who had had a tumour of this kind on the left side of the neck, fully the size of a child's head, which had continued for years, and incommoded him only by its bulk. He refused to submit to its extirpation; and I was informed lately, by a medical student from that place, that he continues in good health, and that the tumour appears to be stationary."

We have seen two or three cases of tumors in the neck, exactly corresponding to the tubercular sarcoma of Mr. Abernethy. They resisted all remedies. One was removed by operation; but the disease returned. Yet the tendency to affections of other organs appears to be much less in this than in instances of genuine medullary disease.

We lately saw a specimen of tubercular sarcoma in the cat. It was brought to the new anatomical school, near St. George's Hospital. We made a section of the tumor, which now forms a preparation in the museum of the school.

We omit two cases—one of medullary sarcoma of the shoulder, in which erysipelas succeeded the operation and proved fatal—the other, adipose sarcoma on the back. We omit these, to proceed to the second part of the report.

### II. On Tumors of the Mamma.

The cases reported are, without exception, instances of malignant disease—medullary sarcoma, or scirrhus. Dr. Macfarlane commences with the candid, and, we think, the judicious admission, that when the whole mamma is diseased, its removal is rarely attended with success. But we do not agree with him in thinking, that when merely a small defined and circumscribed tubercle exists, the operation is much more advantageous. So far as we have learnt from those whose experience has been great, and so far as we have seen in the more limited circle of our own, the extirpation of cancer under any circumstances, and at any period, offers but a gloomy prospect for the patient.

Yet we cordially concur with Dr. Macfarlane, and with all who recommend an early employment of the knife; for, if there be be a chance, it is then; and the surgeon is not justified in throwing it away.

We have already hinted, and we more explicitly repeat, that the faith of some in the cures of malignant disease appears to be ready and capacious. Satisfied with the cicatrization of the wound inflicted by the knife, and with the patient's departure from the hospital, they do not investigate the subsequent occurrences, or, at all events, they do not inform us of them if they learn them. It is this ready method of publishing cures of cancer by an operation, that has led to extensive and pernicious error. It is a practice which cannot be reprobated too strongly, because it is subversive of the best interests of science, of philosophy, and truth. In the report before us, we have three instances of carcinoma of the mamma, attended with enlargement of absorbent glands, and one case of medullary sarcoma of the organ, accompanied with a large tumor in the axilla, in which the operation might be stated to have made a cure. In all the wound healed, and all were dismissed, apparently restored to health. But Dr. Macfarlane very properly indulges in the following train of reflections. Speaking of one of the cases alluded to, he observes—

"The carcinomatous structure of the mamma, as well as of the axillary glands, was distinctly marked,—the disease being in various states of progression. In some places it had the firmness of cartilage, and in others it was soft and friable. When this breaking up of the original tumour takes place, the neighbouring lymphatic glands become speedily involved in the same diseased changes. I have never known an instance in which an operation performed in such circumstances was ultimately successful,—the disease always returning, and that at no distant period. We will even find the same want of success in our operations for carcinoma of the mamma, although the diseased axillary glands bear no resemblance to the texture or appearance of the original tumour. Sometimes these glands are only sympathetically affected; and, without participating in the morbid process going on in the breast, they may altogether disappear. I have seen this happen to enlarged glands, above the clavicle, which appeared to be connected with a scirrhous mamma; but, as this result is comparatively rare, we ought not to rely on the spontaneous disappearance of such tumours, and allow them to remain when we proceed to the extirpation of the disease. I am afraid, therefore, that an operation will be altogether hopeless, when the axillary glands, as in the last three cases, have not only participated in the morbid action, but also in the structural changes of the adjoining disease. It is easy to exhibit, from the records of public hospitals, a lengthened list of successful cures; the individuals having not only recovered from the immediate effects of the operation, but where, for some time after their dismissal, no vestige of the disease can be discovered. That a few of these cases may be cured permanently I am not inclined to deny; but were the whole watched, and accurately traced for a few years, it would be found, in nine cases out of ten, that a return of the disease would take place in the vicinity of the part from which it was extirpated."

We shall only select five cases, and a few incidental remarks. The remainder display, what, alas, is too familiar, the ordinary history of this baleful malady.

Case 8.—*Carcinoma of both Mammæ—Diseased Axillary and Supra Clavicular Glands—Subcutaneous Tubercles.*

Mrs. L. æt. forty-six, and the mother of several children, was admitted on the 1st May, 1826. Both breasts were enlarged, slightly irregular, of a stony hardness, and especially the left one adhered intimately to the parts beneath and to the skin, which was in some parts slightly discoloured, thickened and tuberculated. The left nipple was retracted, and surrounded by a hard scabby areola; and, in addition to a diseased enlargement of the axillary and cervical glands, the integuments of both breasts, as well as those on the front of the thorax and abdomen, as low as the umbilicus, were thickly studded with small, hard, discoloured, and painful tumors, which appeared to be situated in the subcutaneous cellular texture. The disease commenced about a year before, in the glandular substance of both mammæ, at nearly the same time; and after six months' duration, the subcutaneous tubercles began to form. She left the Infirmary in the beginning of June, after having tried a variety of local applications without any benefit. Previous to her death, which occurred in about six months, ulceration of the left mamma, and of a number of the tubercles, took place, and ultimately thoracic disease supervened.

"I have only seen other two cases in which there existed the same tendency to the formation of scirrhous tubercles in the subcutaneous texture,—a combination with carcinoma of the mamma which is not frequently met with. The first patient was an emaciated, unhealthy old man, who, after having had, for several months, a distinct carcinomatous tumour in the left breast, was affected with painful tumours under the skin in different parts of his body, several of which proceeded to ulceration. In the other, about four months after a scirrhous mamma was extirpated, the edges of the cicatrix became indurated, painful and livid; but, before it gave way, subcutaneous tubercles formed, and the disease proved speedily fatal.

The origin of these tubercles, which have all the characters of scirrhus, is to be ascribed to constitutional and not to local causes, as is evident from the fact that they may form during the progress of a carcinomatous mamma in the external surface and in the internal cavities of the body, to which parts it is impossible that the diseased action could have been communicated through the medium of the absorbents. Their existence must be, therefore, considered as contra-indicating the use of the knife, even should the disease of the breast be otherwise favourable."

We have seen a number of subcutaneous tubercles surrounding a scirrhous breast in two instances, and encircling a malignant tumor in the neck in one instance. In none of the patients was an operation ventured on. In one exceedingly remarkable case, which occurred at St. George's Hospital, a patient was covered almost universally with subcutaneous scirrhous tubercles, although no other malignant tumor was discoverable. We are ignorant of the result. We perfectly agree with Dr. Macfarlane in rejecting the knife when these formations have appeared.

Case 9.—*Carcinoma of the Left Mamma — Operation — Dysentery — Death—Scirrhous Tubercle in the Uterus.*

H. M. æt. 51, married, but childless, admitted Aug. 3, 1831. The left mamma was almost converted into an irregular tumor of a stony hardness. The integuments were unaffected. There were three enlarged glands high up in the axilla. The bowels were loose; and the patient had suffered for many years from dyspepsia. On the 7th, at the patient's urgent request, the mamma and diseased glands were removed. Both exhibited a carcinomatous structure. On the 16th, dysentery commenced, and on the following days it was decisively established. On the 19th she died. The mucous membrane of the intestines was not found actually in a state of ulceration; but displayed in different places elevated and ecchymosed patches, with a texture softened and pulpy. There was a small scirrhous tubercle in the substance of the uterus. We subjoin the brief remarks of Dr. Macfarlane.

"From the external condition of this mammary tumour, and from its structure on dissection, it was evidently carcinomatous; and the fact of a tubercle existing at the same time in the body of the uterus, and possessing the same morbid appearances, showed the disease to be constitutional. There was, however, a symptom a-wanting which almost uniformly accompanies this disease, viz. pain. I have only seen another case in which the breast advanced to ulceration, and the disease proved fatal without creating the slightest uneasiness; but there existed in this case severe pains in the arms, legs, and back, which I have frequently observed during the progress of this malignant affection, both when seated in the external parts and in some internal organ. When present, I am inclined to consider them as certainly indicating the existence of a constitutional tendency to the disease; and I have uniformly observed, that should an operation be had recourse to under such a combination, the disease will, at no distant period, show itself in a different part of the body. It constitutes what Sir A. Cooper has not inaptly called cancerous rheumatism."

The chief interest attaching to the case appears to us to be the co-existence of the uterine scirrhus, with that developed in the mamma. The danger to be dreaded at the present day is the performance of unnecessary operations, and the object of those who write for the instruction or amusement of the profession should be to discountenance the barbarous practice. It is on this account we have quoted the judicious opinions of our author, on this account we dwell on the uprofitable subject of malignant tumors, and on this account that we draw attention to this case. At a time when there was no evidence of contamination of other organs than the mamma and the glands of the axilla, the uterus was actually invaded by cancer—an instructive instance of how little worth is local treatment for a malady essentially seated in the constitution.

The next case we shall select is one of scirrhous tubercle, not in the mamma but attached to it. Dr. Macfarlane is of opinion that scirrhus in this form is generally slow in its progress, and more favourable for extirpation than when the gland is itself affected. But to the case.

Case 10.—*Carcinomatous Tubercle of the Left Breast.*

Mrs. S. æt. 50, the mother of three children, admitted August 29, 1831. About a year before, she observed a hard, painful, and circumscribed tumor, about the size of a walnut, attached to the outer edge of the breast, near the axilla. It did not increase, but the paroxysms of pain to which it gave rise gradually became more frequent and severe. There was no disease in the axilla. General health good. Catamenia ceased six years before. On the 18th September, the mamma was removed, and on the 11th of October the patient was dismissed cured.

On inspecting the breast, the tubercle, which was in a scirrhous state, was found firmly attached to the outer edge of the mamma. On making a section of the tumor, condensed fibrous bands could be distinctly traced into the glandular substance of the breast, to a considerable distance from the point of its external attachment.

"I cannot agree," says our author, "with Sir A. Cooper, when he asserts that scirrhous disease of the mamma is most frequently met with in the form of tubercle. My experience, on the contrary, leads me to state, that for one case in which a distinct and well-defined tubercle exists, either in the glandular substance of the breast or connected with it, we shall meet with six or eight cases in which the whole mamma is affected. When the former state exists, the absorbent glands are longer of becoming contaminated, and an operation will prove more successful in completely eradicating the disease. Should extirpation be had recourse to it is the safest practice in all cases of carcinomatous tubercle to remove the whole breast; as, without this, there is a risk of its recurrence, owing to a number of these fibrous bands, so characteristic of this disease, penetrating deeply into the substance of the mamma."

We are disposed to think, with Dr. Macfarlane, that insulated tubercle is not the most frequent form of scirrhus as it attacks the mamma. But we are not so confident of its minor proportion of malignancy. His recommendation to extirpate the whole breast, no matter how small a part of it is involved, is supported by the practice of the most experienced surgeons. Dr. Macfarlane indeed entertains and expresses one or two opinions, which though evidently founded on attentive observation may yet be considered perhaps, somewhat fanciful. He believes, for example, that when there exists, in addition to the usual appearances of carcinoma, one or more cysts filled with bloody fluid, the disease is more actively malignant and seldom if ever eradicated by an operation.

Leaving scirrhus, and neglecting some cases remarkable only for their uniformity, we conclude, without exhausting the subject of malignant diseases of the breast, by mentioning two instances of medullary sarcoma of the organ. This form of cancer is almost invariably more rapid in its progress, and more liable to be complicated with disease of other organs, than scirrhus is observed to be. It is not so frequent in the mamma as the latter, though other tissues, the testis for example, are constantly invaded by it.

Case 11.—*Medullary Sarcoma of the left Mamma, &c.—Wound and Ligature of the Axillary Vein.*

Mrs. B. æt. 49, admitted Sept. 12, 1831. The left mamma was enlarged to the size of the fist, and indurated at the base, but elastic and tuberose on the surface. It was freely moveable, and its upper half, which was the most prominent part, was covered by integuments of a purple colour, which were traversed by varicose veins. A tumour, possessing similar characters, and about the same size, occupied the left axilla, and was so firmly fixed as to admit of but limited motion. From this, acute pains darted along the arm, which, with the pain of the breast, prevented sleep. There was also considerable thickening of the soft parts under the middle of the left clavicle, producing a slight projection perceptible to the eye, but no defined tumor could be felt.—General health good.

When the disease was first observed, about two years before, at which time the catamenia had ceased, it was the size of a filbert; but did not increase much till six months ago, when the tumor began to form in the axilla.

"On the 20th, the diseased parts were extirpated. The removal of the mamma was speedily accomplished; but considerable difficulty was experienced in detaching from the axilla the diseased mass, which was fully larger than the breast. It adhered intimately to the margins of the pectoralis major and latissimus dorsi muscles, to the fascia, to the plexus of nerves, and, for nearly two inches, to the axillary vein. Notwithstanding the utmost caution in separating the tumour, which, from the firmness and intimacy of the adhesions, had to be done with the knife, the axillary vein was unavoidably wounded, and profuse hemorrhage produced. I was averse to include the wounded vein in a ligature, or to thrust a large sponge into the wound, and retain it there till free suppuration was established. In adopting the former plan, there was a risk that the inflammation produced by the ligature might extend along the vein to the heart, and prove fatal; at the same time I considered the latter practice, from what I had seen in another case in which it was adopted, to be uncertain in its effects, and incapable of arresting the hæmorrhage, unless the sponge were secured with a degree of tightness which would interfere with the circulation in the axilla, and be productive of both local and constitutional excitement. It appeared to me that the unfavourable symptoms, from the application of a ligature, might be avoided, by pinching up and tying the wounded part, without including the whole calibre of the vein. This was accordingly done, by transfixing with a tenaculum both sides of the wound in the vein, drawing it out, and passing a ligature around it.

The tumour was then separated from its remaining attachments, and completely removed."

The patient was in a state of syncope, but she recovered, the wound healed, and she was dismissed on the 11th of October. On making an examination of the tumors, they were both decisive specimens of medullary sarcoma. On the 8th of December, the woman died of serous apoplexy at her own house. The cicatrix could then be distinguished with difficulty from the sound skin. The parts in the axilla were condensed and matted together. The axillary vein was pervious, but its calibre was slightly diminished at the point to which the ligature had been applied, No vestige of the fungoid disease could be discovered.

Dr. Macfarlane owns that he was exceedingly averse to the performance of the operation, and was only induced to accede to it by the urgent intreaties of the patient. He says that he has seen several cases in which the diseased parts were amputated at a much earlier stage, and where there was but little, and in some instances no affection of the axillary glands; but the result was uniformly unfortunate. Our own experience perfectly accords with that of our author. Within the last three years we have enjoyed the opportunity of seeing two cases in which ulcerated medullary sarcoma of the breast was removed. In both the wound inflicted by the operation healed. But in one the patient died at the end of two months, with medullary tumors springing from the cranial diploë; and in the other, death ensued in the same time from medullary tumors in the lungs. Such is our experience of this most malignant affection.

Case 12.—*Medullary Sarcoma extending from the Axilla to the Breast.*

M. S. æt. 39, married, admitted Feb. 3, 1832. The right axilla was filled with a hard, irregular, and flattened tumor, of an oblong shape, which was the seat of violent paroxysms of lancinating pain. The fingers could not be insinuated between its upper margin and the axillary vessels: it dipped under the edge of the pectoralis major and latissimus dorsi muscles, and admitted of but limited motion. A thickened band was traced from its inferior border to the mamma, a small portion of which felt hard and painful. She slept ill, and complained of pain, numbness, and inability to move the right arm. The tongue was smooth, and of a dark red colour,—appetite impaired,—bowels natural,—menstruation regular,—countenance sallow,—pulse seventy-two. The tumor in the axilla was first observed three years before, about twenty-one days after she was delivered of her second child. It was then the size of a field bean; but did not increase much, or become painful, till about five months ago.

The tumor seemed to be scirrhus. Our author was disposed to avoid an operation. But the patient was resolved on its performance, and it was performed. It was done on the 12th, the tumor, mamma, and intervening band of thickened substance being extirpated. One of the axillary nerves was deeply imbedded in the tumor, and was divided. On the 10th of March, the patient left the hospital improved in health.

The tumor presented, on dissection, the structure and appearance of medullary sarcoma. It was composed of three cysts; one of which contained a bloody fluid, and the others a soft, yellowish-white, brainy-looking substance, in which were small clots of blood. The inner surface of these cysts had a dark-red villous appearance, and was studded here and there with small spongy granulations, the encephaloid substance in immediate contact with these points being in a state of greater mollescence than in any other part of the tumor. The outer edge of the mamma was hypertrophied and indurated; and in the cellular substance, immediately exterior to it, two enlarged lymphatic glands were found.

Dr. Macfarlane observes the difficulty of distinguishing between scirrhus and medullary sarcoma in this case, a difficulty sometimes felt. In the early stages of the latter much must depend on the quantum of compression to which the tumor is submitted. In general, medullary sarcoma is attended with less pain than scirrhus. This seems to depend in some degree on the greater degree of condensation of the latter. If the former is much straitened and confined by the pressure of fasciæ or of muscles, its texture is rendered firmer, and not unfrequently the lancinating pain of scirrhus is experienced.

Dr. Macfarlane has not touched on an interesting point, the varieties of constitutional contamination observed in cases of scirrhus and medullary sarcoma. The affections of the lungs are perhaps the most frequent, and after them come malignant deposites in the liver. It would be interesting, if not important, to determine, by a careful calculation of cases, the periods at which diseases of other organs supervene, the organs most affected, and the ratio of frequency of their affections. Much time would be required, much experience necessary, to determine extensive facts of this kind, and perhaps we must trust more to isolated cases, and the common mass of common observation, than to any elaborate individual researches.

We had intended to include in the present report the subject, and many cases, of tumors of the abdomen. But its length must compel us to defer the latter to another opportunity, when we shall endeavour to throw together some other facts, in addition to those brought forward by our author.

---

### St. George's Hospital.

### Remarkable Distortion of the Cervical Vertebræ, with Clinical Remarks, by Mr. Cæsar Hawkins.

*Scrofulous Caries & Tumor of the Spine. Cure.*

Harriet Cumming, æt. 10, admitted April 9th, 1834, under the care of Mr. Hawkins, with partial paraplegia of all the extremities, so that she cannot grasp any thing in her hands, nor support her weight on the legs; neither can she direct either limb at once, and freely, to any particular spot, any movement being conducted slowly and with effort, and several attempts are often necessary before the particular action can be performed. The sensibility of the arms is also a little impaired. The head is scarcely capable of being moved at all, in any direction, being impeded by some displacement, and by a tumor around it; the circular motions of the head do not allow of the chin being rotated above two inches, and although she can bend the chin forward so as to touch the chest, she cannot bend it backwards at all; so that when it is as erect as she can make it, the chin is not above two inches from the sternum, and if she moves the head much, it is with the assistance of her hands. The chin is much more forwards, and much more depressed, than natural, and the occiput necessarily more on a level with the back of the neck and with the shoulders than it ought to be. The back of the neck, at the same time, presented a remarkable appearance; since, besides its being shortened and flattened by the approximation of the head to the shoulders, it was enlarged and widened by tumefaction, formed around the spine, which quite prevented the different vertebræ from being distinguished. This swelling was chiefly at the sides of the vertebræ, and partly around the spinous processes; but, as far as could be ascertained from examination in the front of the neck, it did not seem to be formed anterior to the bodies. This swelling was smooth and uniform, and, though elastic, was evidently composed of solid substance. There was no pain on pressure, neither did the movement of the neck seem to occasion any pain. Her health had throughout been good, and there seemed to be only a trifling febrile affection. Bowels open naturally—urine healthy—perfect control over the rectum and bladder.

*History.* The affection is attributed to a blow three years ago, her head having been struck against a ceiling; as far, however, as can be gathered from her mother's account, there may have been some partial loss of power over the upper extremities at the time, or soon after the accident; but she has been going about without pain or inconvenience till about three weeks ago, when she suddenly lost the power of standing, and regulating the movements of either limb. It would seem, too, as if, at that time, the left upper extremity was first partially paralysed, then the right arm, next the right lower extremity, and lastly the left.

In investigating the case, Mr. Hawkins gave it as his opinion that the case was a complication of scrofulous caries of the bones of the spine, with a peculiar deposit of organized substance among the ligaments of the spine. With regard to the caries, the mode in which the destruction of the bones had taken place seemed to him to account for the peculiar deformity. The caries was usually in the *bodies* only of the vertebræ, so that the arches remaining entire, the loss by absorption in front occasioned a sinking in that situation, and, consequently, an angular curvature backwards in the spinous processes, which still remained attached to one another. In this case, however, the *articular processes* also had been destroyed, as well as the body of one, or perhaps of two vertebræ, so that the head, with the four upper vertebræ, had sunk altogether downwards and forwards, occasioning the remarkable circumstance of the chin being actually as low as the sternum, and, in some positions, touching the chest below the upper part of the sternum—while there was, at the same time, much more of the head in front of the spine than there ought to be, the line of the lower vertebræ no longer corresponding with the foramen magnum, but with the back of the occiput. The spinal marrow was thus not merely bent, but must be, to a certain extent, bent doubly, so as to describe a deviation from its proper course resembling the letter S, and yet without causing much paralysis. This circumstance, Mr. Hawkins observed, is sufficient to shew that, in disease of the spine, it is not the pressure upon the spine, but the irritation propagated to the membranes and substance of the spinal marrow, which occasions the paralysis. A very great curvature may, therefore, allow a person to walk about firmly; and a case in which there is no curvature whatever may be attended with the most complete loss of power if there is inflammation close to the spinal marrow; and this girl, though paralysed now, may perhaps recover the use of her limbs, though the distortion will remain as great as ever.

For the same reasons, the muscular movements only of the extremities are usually impaired; because, the caries being confined to the bodies of the vertebræ, the irritation is propagated only to the anterior part of the spinal marrow, on which those movements depend, while the posterior part remains unaffected. In this case there is a little loss of sensibility, not, perhaps, from the posterior part of the spinal marrow being a little inflamed (though this is of course possible, when the caries affects the oblique processes as well as the bodies), but probably from the tumor around affecting the cervical nerves. I judge that this is the case, because the upper extremities only are partially deprived of sensibility, whereas, if this arose from an affection of the spinal marrow, it should of course be observed in the legs also, just as you observe it in injuries of the spinal marrow, along with the other fatal effects upon the parts below the injury, which arise from the loss of the important vital functions, which depend upon the influence of the posterior part of the spinal marrow.

The case before us, in which there is an entire absence of pain, will serve also to shew the *scrofulous* nature of the caries, as distinguished from ulceration of the intervertebral substance in adults; there being, in these cases, exactly the same distinction that may be often observed, in the hip-joint, between the scrofulous affection of the head of the femur and the primary ulceration of the cartilages. The tumor formed round the vertebræ is of a peculiar nature, which I have seen in several instances, and which may occur independent of caries of the bones. I have seen it in a child, after a blister had been applied to the neck for irritation of the brain, occasioning complete stiffness of the neck, with partial paraplegia, and with the same flattening and widening of the neck as in this child. I have seen it also, however, in adults, following injury of the neck, where there was no reason to suppose that it was scrofulous matter, and where it disappeared under blisters. In fact, in this girl it appears to be more solid than scrofulous matter usually is; and in a preparation in the museum, you may see that, in a case where it followed exposure to cold, and was ultimately fatal, there is a deposit which is completely organised, which scrofulous deposit is not, and has filled every part around the spine, and passes in by the foramina into the vertebral canal, where it is situated all round the spinal marrow. In a young man in whom there was a swelling of this kind, the deposit seemed to be around the sides of the vertebræ only, and external to the spinal canal, and the upper extremities were paralyzed without any affection of the lower limbs; and the swelling was in great measure dispersed by repeated blisters, and the motion nearly restored to the arms. The indication, in this case, appears to be to check the caries, and procure absorption of the deposit around by blisters, and perhaps some benefit may be derived from mercurial ointment, as a dressing to the blister.

April 10th. *Empl. lyttæ nuchæ, et Ung. hydrarg. fort ulceri.*

12th. *Olei ricini,* ℥ss.

15th. *Rep. Ol. ricini.*

19th. *Rep. Empl. lyttæ, et Ung. hyd. fort.*

22d. Has a little more power over the limbs. Swelling rather lessened.

24th. *Haust. rhei.*

26th. *Repr. Empl. lyttæ, et Ung. hydrarg.*

28th. ℞. *Calomel,* gr. iij. *Pulv. rhei,* ℈j. *statim.*

May 4th. *Rep. Empl. lyttæ, et Ung. hydrarg.*

13th. More power over the limbs; she is able to stand and support her weight, and to move a little without other assistance than a table or the bed. The swelling, though still considerable, is firmer, and more divided into several portions. Health improved, so that she has gained flesh since her admission. Allowed to sit up in a chair.

20th. *Repr. Empl. lyttæ, et Ung. hydrarg.*

24th. Can walk slowly and feebly indeed, but without holding by any thing.

June 3d. *Rep. Emp. lyttæ, et Ung.*

21st. Discharged as cured, being now able to grasp firmly, and to walk tolerably firmly and quickly. She can raise her head much more, though she is unable to bend it backwards; she can also rotate the head considerably more, and is not now afraid to do so quickly. The appearance of the deformity is very striking from the chin, when the head is much raised, being so much before the sternum, and nearly on a level with it.

Dec. 1834. This girl has frequently been to the hospital to shew herself to Mr. Hawkins, and she has remained quite free from any complaint, with tolerable freedom of motion in the neck, except backwards, in consequence of the distance between the points of the spinous processes of the fourth and fifth vertebræ from each other, the upper one necessarily impeding the backward movement of the head, though allowing of motion in every other direction.

---

### Liverpool Ophthalmic Infirmary.

### Report of the Practice for the Year 1834.

This Report is contained in a lean octavo volume, published by Hugh Neill, surgeon to the charity. This gentleman's enthusiasm in the cause of ophthalmic surgery appears to be of no common order. He quotes, and he quotes with rapture, the boast of M. Bourgot St. Hilaire, that he, M. St. Hilaire, was a surgeon-ophthalmologist, or what is profanely denominated an eye-doctor. What can be more noble, what can be more French, than the ophthalmologist's apostrophe.

"Naturaliste aux écoles publiques, au Muséum, je me ferai gloire de rester aux yeux du corps médical, médecin et chirurgien ophthalmologiste!"

But, as Mr. Neill says, enough of this. When we descend from the elevated style of the preface to the sober groundwork of the report, we find comparatively little to detain us. Yet the report is spread through fifty-five pages, and more might have been given, both of interest and instruction, by one as much devoted to ophthalmic surgery as Mr. Neill would appear to be. The subject which attracts most of the attention of our author, and which occupies the greatest space in the report is the use of strychnine in amaurosis. Our readers must be aware that this formidable remedy has been highly vaunted in this formidable disease, and that many have found, or have said they found it almost a specific. There is no profession, nor any portion of human learning in which experience teaches us more sadly to doubt and disbelieve what is said and what is written, than it does in our's. The success that we read of we seldom see, and the critic has constantly occasion to remark, that means precisely opposite succeed in the hands of different individuals, to the exclusion of every method but their own. If men do not lie, truth is contradictory, and in order to believe we are too often called on to evince much charity and little judgment. We will not say that scepticism, we are talking of our science only, we will not say that scepticism is generally to be recommended, but the rational philosophical spirit of doubt must be entertained by every man of sense. Enthusiasm perverts as much as knavery, nay, it is more dangerous, because it is received with less suspicion of dishonesty. In medicine there is at least as much room for self-delusion as for voluntary fraud, and who has not observed in his professional walk, the innocent impostor, convincing himself and striving to persuade others, that facts are all in favour of his visionary perhaps ridiculous opinions. But we drop this train of general remark, excited by the connexion of strychnine and amaurosis, and proceed to Mr. Neill's observations.

### AMAUROSIS.

"My practice," he says, "has been general. I have cupped, purged, and depleted where plethora was evidenced. I have stimulated where there was atony; and where general debility has shown itself, I have not been deficient in the exhibition of my tonics. Mercurials, too, have had their fair trial; but I have not *yet* found a *panacea*.

If any one remedy has stood my friend more than another, it has been *Strychnine*, judiciously administered, combined, or not, as necessity pointed out, with one or other of the general forms of treatment already alluded to.

Surgical assistance, in some cases, is unfortunately of no avail; but in the early stages of the complaint, the skilful practitioner may, and *can*, do much to protract, if not to prevent, its progress; and the disease, if not cured, may be made to linger on its way."

This is not injudicious. Mr. Neill does not vaunt the drug too highly. He observes in continuation:—

"In no instance have I seen Strychnine useful in Amaurosis, if internally administered; nor in any have I seen it successfully used where the *Iris* had completely lost its motion. In many cases also, where I have used it with advantage, no twitchings were perceived by the patient; but just about the time that sight began to improve, a very considerable sensation of bitterness was communicated to the palate, a few hours after each application of the remedy.

It is also but justice to acknowledge, that I have often been disappointed in the use of Strychnine, in cases where I had looked for the happiest results."

Mr. Neill quotes seven cases from a paper which he published in the second number of the Liverpool Medical Journal. That paper was noticed in several other journals, and we need not therefore resuscitate its contents. But a new case is added to those before published, and this one we will introduce. It is an instance of paralysis of the rectus superior oculi. We will give it in our author's words.

"I was lately consulted by a young and lovely lady, who for six years had been afflicted with symptoms which rendered her existence almost miserable. About six years ago, she had suffered from severe rheumatic fever, aud was for many weeks confined to a sick bed. Upon her recovery, it was discovered that the superior straight muscle of the right eyeball had lost its power. Headach followed the rheumatic attack; these headachs became more and more frequent—the lids began to fall—sight became diminished, with slight outward squint. Medical men after medical men were consulted, but the ailment became more troublesome. Several of the Metropolitan opinions were taken, and their advice pursued, but with no advantage.

I found her seated with the lips drooping. If she wished to raise her eyes as far as my face, her chin was required to be thrown up, until the effort and effect were most painful. The right eye had lost its brilliancy; it was dull and muddy looking. She complained of great weight over the brows, and of general debility; and expressed her determination to endure any pain or uneasiness, provided the slightest hope could be held out of even partial recovery.

I proposed the use of Strychnine, by blister to the temple, and immediately commenced its application.

A small *horse-shoe* blister was applied over the right brow, at night; and on the following morning the one-fourth of a grain of Strychnine was dusted over the denuded surface. On the following morning, half-a-grain. Next morning, the same quantity. Fourth morning, a grain. On the evening of this day, the lady said that 'she thought, when she looked at a printed page, the type and paper were brighter than they had been.' Fifth morning, a grain. She is certain, this evening, 'that *there is* improvement.' Her spirits are high—no twitchings—tongue clean.

On the seventh day, a grain is applied morning and evening. She slept badly, started much during the *night.*

On the eighth morning, one grain and a half is applied. About five o'clock, P. M. she was seized with difficulty of breathing—general languor—cold feet—her face was flushed. She had hot bottles to the feet, and stimulants; this difficulty of breathing wore off about eleven o'clock at night—she slept well.

Ninth morning, the lids are *well up*—the eye's motions restored—it rolls rapidly in every direction, so far that the cornea is raised at its lower edge above the edge of the lower lid.

There is, however, unequal motion when the two eyes act at the same time. I continued the Strychnine for five days longer, in the quantity of one grain every morning, and the improvement gradually progressed. Fourteen grains in all of Strychnine were applied.

I now commenced the use of tonics, with small doses of blue-pill; one grain each night. The sound eye is bound up, and the (formerly) bad one is obliged to do all the work; it is *bright, lively, and sparkling,* but has not obtained complete motion upwards.*

The two eyes are parallel until they catch the cornice of the ceiling. But upon a sudden exertion, or in straining the eye much upwards, there still is to be perceived a slight want of parallelism. However, I am not without hopes that this imperfection will yet be overcome.

All dropping of the lids, all imperfect sight, and general uneasiness, from a sense of weight over the forehead and brows, are gone.

I must not omit mentioning, that when I first saw the lady, the pupil was dilated and the iris sluggish; indeed motion was almost lost. But after the fourth application of the Strychnine, it gained the most lively motion."

It cannot be concealed that the report, if considered final, is somewhat premature. Three weeks only have elapsed since the last application of the strychnine. Yet with this slight drawback, the case deserves attention.

Dr. Ryan and Mr. Neill have found strychnine useful in some instances of deafness. Yet the fact on which Mr. Neill founds his faith is but a lame one.

*Case.* " Mrs. H., aged 50, after the birth of her first child, was seized with deafness in both ears. She has never had inflammation in, nor discharge from them. This deafness has now constantly existed for thirty years, and is so complete as to require the con-

---

* " The reader will understand what I mean by 'complete motion,' when I mention the fact of this lady being now able to exercise her eye, and improve her bodily health, by daily amusement at the billiard table."

tinued use of a trumpet. She says that sounds are *heard* with one ear, but are *perceived* with the other. *One* hears the sound, the *other* distinguishes (internally) what that sound is. She consented, on the 14th April last, to have a blister applied behind the right ear, and on the following morning I commmenced the use of strychnine, by applying half a grain daily over the blistered surface. This was continued for three days, when the quantity was increased to one grain. On the fifth day, she heard *distinctly* without her trumpet, which she laid aside; on the sixth day, sounds the most minute were distinctly heard, and sharp sounds were extremely painful; on the evening of this day, she complained of lowness of spirits; she did not sleep at night. On the seventh day, she frequently burst into tears, although she could not say from what cause. The strychnine was discontinued. For four days longer her hearing was retained. As the nervous agitation decreased, her deafness returned, and on the fourteenth day from the commencement her deafness was as complete as before.

I waited a week, and again applied a blister behind the left ear, and commenced the use of strychnine as in the former instance. The same effects were exhibited until the sixth day, when I speedily resigned the use of the remedy, from the patient complaining of numbness and dragging in one leg. Her deafness returned in a few days. She can now, as formerly, only distinguish the sounds of the voice by having her trumpet held in proximity to the speaker."

### CATARACT.

Experience is dogmatical, and so, being experienced, is Mr. Neill. He thus decides his, the best, operation for cataract.

"My operation is the needle operation, and my reasons are—

1st. For *Congenital* Cataract, no man should use any other instrument than a needle.

2d. For *soft* Cataract, it would be unsurgical to attempt any other than a needle operation.

3d. For *Capsular* Cataract, when the lens has ceased to exist, or is softened in texture, the needle is the best instrument.

And lastly. For *hard* Cataract, the *reclination* operation by the NEEDLE is surgical, elegant, least painful, and less likely to be injurious than any other operation.

You should not extract a Congenital Cataract; you should not *attempt* to extract a *soft* one; and you *need* not extract a hard Cataract."

### CONTUSIONS AND WOUNDS OF THE EYE.

Mr. Neill remarks that, in these injuries, prevention of inflammation is the paramount object. "I may contrast," he says, "several cases. One was a fine young woman, from Cheshire, who came to me with general suppuration of the eye-ball, resulting from a slight scratch of a thorn on the cornea; her eye was lost from want of timely aid. I may contrast this with two other instances, where injury more severe occurred, and yet sight was not affected. One had a penknife run through the cornea, and the protruding portion of iris was strangulated for two hours before I returned it; the other, the eye-ball was transfixed with a fork; and yet in neither of these cases was sight injured. But I could repeat a multiplicity of cases in proof of this point. There is such a difference in general surgery. You there look for reaction as a healthy process; and when it comes, you temper it. In the EYE, however, you ought to dread, and should prevent reaction. Inflammation, long before it gives notice by PAIN, has done irreparable injury to the non-sensitive and delicate structure of the INNER eye."

Mr. Neill dwells long on the reputed sensibility of the surface of the eye. That sensibility he thinks, with Mr. Guthrie, greatly over-rated. Yet he differs from Mr. G., in not granting, as the latter does, an equal amount to the lid and eye-ball.

"I grant," he says, "that if dust lie gently on the lining membrane of the lid, it will not produce pain; but if you press it upon that membrane, very acute pain will instantly be produced, accompanied by lacrymation, and distended vessels; showing a sympathetic and easily excited sensibility in the contiguous texture. But you may press a body, rough and sharp, against the eyeball, without much uneasiness; and further—you may, as I do every day, touch the corneal or sclerotical conjunctiva with the solid nitrate of silver, and the patient does not exclaim that he suffers pain, *until* the lids are allowed to glide over the injured surface, and THEN, how acute it is! But touch the *lining membrane* of the LIDS with nitrate of silver, and pain is the immediate result!

Hence I infer that that sensibility to pain which has usually of late been attributed to the eyeball, alone pertains to the lid.—Observe, I speak of the eye-ball in health; for when inflammation has attacked the compact texture of the tunics, we all know how sensitive they become.

The conjunctiva of the eyeball is comparatively insensible: it is braced on the ball tight as a drum head. Draw the skin tight over a healthy finger, and you may prick it, but pain is scarcely perceived."

We cannot exactly agree with Mr. Neill that the conjunctiva is stretched upon the eye-ball as tight as a drum-head. The conjunctiva covering the sclerotica is loose, and, *if* conjunctiva does cover the cornea, it is at least much modified in structure. Under any circumstances it is the acmé of absurdity to suppose that it is stretched so tight, as by the strangulation of its vessels, to be insensible to pain. The idea is quite preposterous.

Mr. Neill is an advocate for belladonna, even during the inflammatory condition of the eye, when the result of injury. He applies the extract over the upper lid and brow.

### Pustules of the Cornea.

" Pustules of the Cornea constitute a dangerous complaint.

There is generally febrile excitement, full pulse, foul or unnaturally red tongue, redly inflamed inferior palpebral conjunctiva, and, in young persons, a round drum-sounding belly.

I purge, bleed by leechings or small cuppings, sicken by the tartarised antimony. Give Dover's Powder at night, use fomentations of poppy heads, and when the irritation marked by intolerance of light subsides, I use the weak solution of nitrate of silver. I recommend the patient to drink water impregnated with carbonate of soda, and finish off with alternate doses of blue-pill.

This complaint sometimes leaves permanent specks. If the Pustule occur in early life, I generally commence my treatment by applying the two grain drop of the nitrate of silver, and give half-grain doses of calomel every night, with antacids."

### Strumous Corneitis.

" This is a very teasing complaint. In the first instance, it is necessary to examine the upper lid; perhaps you may find the vascularity of the Cornea produced by tubercular granulations on the lining membrane of the lid, which mechanically keep up constant irritation. If so, they must be touched with sulphate of copper or nitrate of silver, and the complaint, by mild treatment, will be overcome.

If the vascularity exist without this irritation, foment, give blue pill, and from time to time apply Mr. Guthrie's ten grain ointment. The *Proto-iod. Hyd.* is, as far as I can judge, a very uncertain remedy; it is a bad and inefficient substitute for either calomel or blue-pill."

Mr. Neill quotes a case of this disease to illustrate the treatment.

*Case.* Margaret Porter, aged 14, from Ormskirk, was admitted an in-patient on the 29th March, 1834.

She has been unable to bear the slightest light for three months. On examining the eye, the Cornea has a bright red appearance, the pupil is not to be seen. Ordered fomentations of poppy heads, three grains of blue-pill night and morning.

April 8th. Pain and intolerance of light gone. The eye can be easily examined, and the Cornea is quite a network of twisted red vessels. Apply Guthrie's *ten grain Nitrate of Silver* ointment, and continue the blue pill at night. The ointment to be repeated twice every week.

May 4th. Made out-patient.

July 8th. No vestige of disease remaining."

### FISTULA LACHRYMALIS.

The only observation on this affection worth quoting refers to a catgut probe of our ophthalmologist's construction. "If there be," he says, "disease of the *os unguis*, as often happens, puffiness will exist around the mouth of the wound, and unhealthy granulations *within* it. In such cases, I use a catgut stilette; it is light, and does not hurt the diseased parts. The catgut swells gradually, and enlarges the opening without producing pain. In fact, under peculiar circumstances, it is an excellent little instrument. One thing, however, must be remembered—it must on no account remain longer than twenty-four hours in the passage. Any surgeon may make it in the following manner:—Cut about an inch of cat-gut bougie; file down one end; give it roundness, and a little tapering; then polish it with sand-paper. To give it a head, hold the cut end to a candle, it will swell, and a little heated black sealing-wax will then give it a famous head. Before the instrument is used, it should be dipped in oil."

We really see nothing else in this hungry volume to detain us. It is a good illustration of the old adage—"much cry and little wool."

---

### CLINICAL LECTURES ON HÆMORRHOIDS.* By Sir B. C. BRODIE, Bart.

The opinions of Sir Benjamin Brodie on points connected with the practice of surgery, are anxiously sought, and, of course, are highly appreciated by the profession. His accuracy of observation, extensive opportunities, and judicious improvements on ordinary treatment, give a value to his lectures on familiar subjects of which they might scarcely be supposed susceptible. It might appear that the history and treatment of hæmorrhoids were sufficiently exhausted. But experience distinguishes many circumstances that have either been imperfectly noticed before, or, if noticed, imperfectly described. And this it is which makes the observations of a practical man, in a science such as our's, more useful to the student than the elaborate information collected by professed authors from every possible source.

Sir Benjamin occupies two clinical lectures with the common disease—hæmorrhoids or piles. Of these lectures we shall present the principal portion. The closely-woven tissue of Sir Benjamin's style of lecturing and writing usually precludes much useful abbreviation.

The distinction of external and internal piles, and the nature of the hæmorrhoidal swelling, first occupy the attention of the lecturer. The former we think we may omit; of the latter we need only say that Sir Benjamin cannot doubt that piles are in the first instance dilated varicose veins. Dissection fully appears to prove this. Here, as in other cases, surgeons have forgotten that, to ascertain the real nature of any organic alteration, the examination should be made at an early period; when this has become advanced, other changes supervene to modify and obscure the first.

"Those ultimate changes," Sir Benjamin remarks, "which take place in cases of piles, are exactly similar to those which occur in connexion with varicose veins of the leg. You know that at first the veins of the leg are simply varicose, or dilated; that at last they become inflamed; that lymph is deposited in the cellular membrane surrounding them, and that at last there is a great mass of induration, in which the diseased blood-vessels are, as it

* Med. Gaz. Feb. 21, and Mar. 44, 1835.

were, imbedded. So it is with the veins of the anus and rectum. At first they become simply dilated; repeated attacks of inflammation cause an effusion of lymph into the adjacent cellular texture, and then the pile appears like a solid tumor; in the centre of which, however, you still find the dilated vein in which the disease originated."

Sir Benjamin observes by way of parenthesis, that though internal and external piles deserve separate consideration, they are in fact affections of the same veins; the action of the sphincter preventing dilatation in the intermediate points. The causes of piles are multifarious, though probably all act in the same immediate manner, by preventing the proper return of blood from the inferior mesenteric vein. Diseased liver, tumors of the abdomen, pregnancy, costiveness are all familiar agents in producing hæmorrhoids.

"Piles are more frequent in the upper classes of society than in the lower. You know that in hospital practice you see comparatively few cases of piles, but out of it, I must say they form a very large proportion of the cases that come under my care. The reason of this difference is to be found in the different mode of life in the various classes of society. The better classes take but little exercise, and they are more liable to constipated bowels than the lower classes, who take much exercise and live a great deal in the open air. There is a notion that those who take aloetic purgatives are more liable to piles than others; but I must acknowledge that I am not quite satisfied of the fact. I have a respect for all popular notions, believing that there is in general some truth at the bottom; and I will not say, as every body thinks so, that aloes will not make people liable to piles, but I am sure they do not produce that effect to the extent that is supposed; and I could not be certain, from my own observation, that they are productive of it at all. The fact is, that those who are habitually taking aloetic purgatives are persons with costive bowels, who, as I have already mentioned, are just the individuals most liable to this disease."

The symptoms of internal and of external hæmorrhoids are different, and they also vary with the stage of the disease. Itching is an early and a well-known symptom; occasional attacks of inflammation both of internal and external piles are also commonly known to take place. Every now and then, says Sir Benjamin, when the patient is costive, the external piles become swollen and tender; the internal piles become swollen also, so as to fill up the cavity of the gut, thus exciting a sensation as though a stick, or some other foreign body, were lodged in it. The external piles sometimes inflame, swell, and become tender, so that the patient can scarcely bear them to be touched, and cannot walk without difficulty. They may continue thus inflamed for some considerable time, and then the inflammation may subside; the piles generally returning to the condition in which they were before the attack of inflammation came on, but not always.

Sometimes an abscess forms in one of these inflamed external piles, and bursts externally. The abscess may be troublesome to heal, but when it is healed it is found that the cavity of the vein is obliterated, and that it is, in fact, cured.

"Such an abscess, as I have just mentioned," he proceeds, "must be distinguished from a *fistula in ano;* from which, indeed, it is essentially different, as I shall explain more fully hereafter. Sometimes, when an external pile is inflamed, the blood in it becomes coagulated, and it is then hard to the touch. If under these circumstances you slit open the pile with a lancet, there comes out a mass of hard coagulum, perhaps as large as a pea or a horse-bean; the cavity inflames, suppurates, and granulates; the same thing happens as though suppuration had taken place in the first instance, and the pile is obliterated. But if you do not slit open the pile, and leave the disease to take its own course, the cavity being blocked up by the coagulum, the vein becomes obliterated, after which the coagulum is gradually absorbed, and the pile is cured; that which was a pile before being now converted into a flap of skin. Just the same circumstance happens with varicose veins of the leg, where sometimes there is a natural cure, in consequence of the coagulation of blood in the dilated vessels. Sometimes, when a pile is thus distended with coagulated blood, the skin becomes so much attenuated that it gives way in some one point, and the blood being gradually squeezed out, suppuration probably takes place; and the case proceeds just the same as if you had opened the pile with a lancet. It is very common for external piles to undergo a process of natural cure in one or other of the ways which I have now described; and by examining the parts, you may ascertain whether these changes have taken place, as every one of them, after the cure is effected, becomes at last converted into a fold or flap of skin. Thus, if you see a patient with three or four loose folds of skin at the margin of the anus, you may know that these were formerly piles. At first these folds of skin are large, loose, and pendulous, but gradually they become contracted, till at last they give no sort of inconvenience to the patient."

Sometimes internal piles are so much distended, that the gut is incapable of containing them. They are then pushed out through the anus and form a tumor, which projects externally, although still covered by the mucous membrane. The protrusion of internal piles gives rise to many observations from the lecturer. When large, they always protrude when the patient goes to the water-closet, and afterwards go up spontaneously. If they be larger still, after going to the water-closet they will not return spontaneously, but the patient is under the necessity of pushing them back with his hand. If they be larger still, they come down at other times, especially when the patient is walking, so that he cannot well take any exercise. Sometimes we see one small internal pile permanently protruded, forming a red vascular tumor of the size of the extremity of the little finger. This is painful, and otherwise very troublesome, to the patient, by keeping up a great and constant discharge of mucus. Sometimes there is a large protrusion of internal piles for several days, then they gradually become reduced in size, and go back into their proper place above the sphincter muscle. In short, with respect to the protrusion of internal piles, there are all possible varieties of circumstances: they may protrude occasionally, for a short time, or for a long period; they may be constantly protruded; or there may be a large protrusion at one time, and a small constant protrusion besides. Whenever the protrusion, be it large or small, takes place, there is an abundant secretion of mucus from the rectum; the piles themselves are sore to the touch; the surface is red and vascular; and if you put your hand upon them, you find that you can diminish their size by pressure, but the moment you take off the pressure, they are as large as ever. Sir Benjamin is careful to discriminate this state from that of true prolapsus of the rectum, with which, however, it is constantly confounded. In the latter the gut itself comes down, perhaps to the extent of several inches. But when internal hæmorrhoids protrude, that portion only of the mucous membrane which covers them descends; as, indeed, it necessarily must. The distinction between the cases is important, and should not be lost sight of, as it is.

Internal piles in this condition produce great inconvenience. Sometimes by irritating the contiguous parts they occasion frequent desire to make water, or even actual retention of urine. They may also bleed freely, whence their name of hæmorrhoids. The blood that flows is usually arterial, and the disposition to hæmorrhage is in the latter stage of the disease, when an increased determination of blood takes place to the mucous membrane and cellular tissue by which the piles are surrounded. The amount of hæmorrhage varies from a mere tinge, to the loss of six or eight ounces daily. When such quantities are voided the usual results of long continued bleeding may ensue.

"Inflammation sometimes takes place in internal piles, and ends in suppuration. The patient complains of a little discharge of matter from the anus, and you find, in addition to the mucus, that there is a little yellow stain of pus on his linen; and at first you would suppose there was a common abscess about the rectum, such as produces a *fistula in ano*. But if you introduce your finger into the rectum, you feel a small orifice in one of the internal piles, and if you pass a probe with a light hand, it goes to the bottom of the abscess, which is perhaps a quarter of an inch in depth, or thereabout. The parietes of the abscess, however, are very thin and weak, easily broken down, and if the probe be not lightly introduced, it will run through them into the loose cellular texture external to the mucous membrane. The cellular texture also is very loose and yielding, offering scarcely any resistance to the probe, so that it will run in every direction; and hence it is that I have sometimes known a small abscess or an internal pile to have been mistaken for a very long sinus. You ought to be very careful not to fall into this error, which you might easily do—nay, in all probability would do—in the first case of the kind that occurred to you, if I did not give you this caution."

Sir Benjamin Brodie has already described the natural cure of external piles—inflammation—consolidation—and subsequent absorption of the lymph. There is a natural cure of internal piles also, mortification.

"Where piles of a large size protrude, completely filling up the orifice of the anus, the sphincter muscle is contracted upon them like a ligature, and causes them to become more swollen than when they were first protruded; just as a ligature on the arm makes the veins of the fore-arm and hand turgid previously to venesection. But the piles may be larger still; the sphincter muscle may contract more powerfully upon them; and then the pressure not only interferes with the return of the venous blood from the pile, but prevents the entrance of arterial blood into it. It acts as a ligature acts in a surgical operation—on a polypus of the uterus, for example. There is not a sufficient circulation in the protruded piles for them to retain their vitality; mortification takes place, sloughing follows, and thus the piles are destroyed. I have known several cases cured in this manner, and there is little or no danger in the process. I have sometimes known medical men to be alarmed at a case of this kind, confounding it with those of mortification from other causes; but the alarm is without foundation. The late Dr. Pearson, who was for a very long period of time physician to this hospital, was the physician and friend of the celebrated Mr. Horne Tooke. Many years ago I was dining with Dr. Pearson, and after dinner he gave an account of Horne Tooke's illness. He said that he had long laboured under piles; that at last mortification had taken place; that there was no chance of his recovery; and he added that he had that morning seen him for the last time. I remember that in the middle of this history there came a knock at the door, on which Dr. Pearson said, 'Here is a messenger with an account of my poor friend's death.' However, it was some other message; but by and by a messenger did arrive, saying that Horne Tooke was much the same, or a little better. It turned out, as I have been informed, that the piles sloughed off, and that from this time he never had any bad symptom. In fact he was, if I have been rightly informed, cured of a disease which had been the misery of his life for many years preceding, and he lived for some years afterwards."

Sir Benjamin Brodie proceeds to the treatment, independent of an operation, before he touches on the mode of operating.

In the early stage mild aperients, especially the electuary of senna, sulphur, and mel rosæ—moderate exercise and diet—lavements of cold water, or water made more astringent with alum, or with the tinctura ferri muriatis, or lime water—such are the familiar items of treatment which most practitioners commonly employ. Sir Benjamin is a zealous advocate of the celebrated Ward's paste. He has often found it highly serviceable, gentle and occasional aperients being combined with it. A piece of the size of a nutmeg should be taken thrice daily, and it ought to be persevered in for two, three, or four months consecutively.

"How (asks Sir Benjamin) does the Ward's paste operate? I know a case in which a patient, labouring under stricture of the rectum, had indiscreetly taken an immense quantity of Ward's paste, and in which the colon was found quite full of it after death. It is evident, that, except any small portion which may be digested, the Ward's paste passes into the colon, and that it must become blended with the fæces; and I suspect that thus coming in contact with the piles, it acts upon them as a local application; much as *vinum opii* would act upon the vessels of the conjunctiva in chronic ophthalmia.

In confirmation of this view of the *modus operandi* of Ward's paste, I may mention an observation of the late Sir Everard Home. He had a patient labouring under piles, and he recommended him to take Ward's paste. The patient, little thinking that something put into the stomach was to cure disease in the rectum, crammed as much as he could bear of it up the rectum. I dare say it gave him a great deal of inconvenience, but, as Sir Everard Home reported, it cured him; and Sir Everard said that since then he had used it as a local application in some other cases, with manifest advantage."

Sir Benjamin has also given with advantage a scruple of the cubebs pepper thrice daily. In some cases, too, he says, where there is a great deal of irritation the patient will derive benefit from copaiva combined with caustic alkali; half a drachm of balsam of copaiva, with fifteen drops of *liquor potassæ,* may be rubbed down with two or three drachms of mucilage and cinnamon water, and taken three times a day. This answers a very good purpose, soothing the piles, and keeping the bowels gently open at the same time.

If called to the patient when the external piles are inflamed and swollen, the surgeon should enjoin rest in the horizontal posture, and apply some leeches in the neighbourhood. If placed upon the piles themselves, the leech-bites are disposed to fester. If the piles are much distended, they may be punctured with a needle, which affords immediate relief, and does not give rise to festering. The piles should be punctured in several places, and a piece of rag wetted with some cooling lotion should be constantly kept upon the part. The patient should also take some cooling aperient.

"When internal piles are inflamed, swollen, and protruded, you should try first of all to push them back into the gut. Take a cambric handkerchief, or a soft old linen rag, squeeze out the blood from the piles, and, if you can return them into the bowel, it is so much the better; it will relieve the patient very considerably. But if you cannot push them up, or if when pushed up they immediately come down again, you should then keep the parts wet with a rag bathed with a cooling lotion, let the patient remain in the horizontal posture, and keep the bowels gently open, without purging. Here also, as in the case of external piles, the patient will derive much benefit from acupuncture in several places. Punctures made with a needle, neither on this or any other occasion, so far as I know, occasion inflammation or any other inconvenience; they evacuate the blood, relieve the tension and swelling, and do a great deal of good without any harm."

The second lecture, for we now quit the first, is dedicated to the description of the operation required for piles, and to a notice of prolapsus of the rectum, and of excrescences of the gut.

### Operation.

Sir Benjamin alludes to the discrepancy of opinion that has existed on the adoption of the ligature or excision for internal piles. Formerly very able surgeons, for example Mr. Cline, removed internal piles by excision. But so many instances have been recorded of dangerous, nay, even of fatal hæmorrhage, in consequence of this proceeding, that most surgeons have been frightened into operating with the ligature. Sir B. Brodie explains the sentiments and follows the practice of Sir Everard Home, both of which he was taught when a student in the hospital. Those sentiments of Sir Everard's are thus laid down :—That external piles which are covered by the skin ought not to be removed by ligature; if they are removed at all, it ought to be by excision. On the other hand, internal piles which are covered by the mucous membranes, ought, *for the most part* to be removed by ligature. In short, the ligature is applicable generally in cases of internal piles, and excision to those which are external. The grounds of this distinction are as follow : The application of a ligature to external piles gives the patient extraordinary pain at the time, and afterwards excites much inflammation, swelling, and disturbance of the general system ; whereas, if they be removed by excision, these ill consequences are avoided. After the excision of *external* piles there can be no danger of hæmorrhage, because the parts are entirely within your reach, so that the bleeding vessels can be easily secured ; and though some little inflammation may supervene on the operation, yet it is not sufficient to be of any real consequence. If, however, you remove large *internal* piles by excision, there may be copious and even dangerous hæmorrhage, since the parts which bleed are out of reach, above the sphincter muscle, where you cannot expose the cut surface, so as to be enabled to take up the bleeding vessel. On the other hand, the application of a ligature to internal piles in general causes but little pain, and only a slight degree of inflammation follows, for the mucous membrane has nothing like the sensibility of the skin, and does not resent an injury in the same manner. With respect to internal piles, then, there is no objection to the use of the ligature, while there is the greatest objection to their simple excision. Sir Benjamin, then Mr. Brodie, was at one time seduced by Mr. Cline's recommendation, and removed internal piles by excision. In the first one or two cases, he found no inconvenience arise from his altered practice; but then a case occurred in which the patient lost a great deal of blood ; in another case, the hæmorrhage was so great that the patient nearly died ; and then a third case occurred, in which also the patient lost an enormous quantity of blood, and he now wonders that he did not actually die. Since that time, Sir Benjamin has never removed large internal piles except by ligature.

" The removal of external piles is very seldom necessary: they are generally complicated with internal piles ; and if you cure the former, the latter, which are a continuation of the same veins, will be cured also. However, there are cases in which it is right to remove external piles by incision. For example, where they are enlarged and inflamed, so that it will take a great deal of time to subdue the inflammation, and the patient is all the while suffering pain, he may be relieved at once by two or three snips of curved knife-edged scissars. Or if an abscess has formed in an external pile, which bursts, discharges, and closes at the orifice, then bursts and discharges again, it may be woıth while to cut off the pile and the abscess with it."

To remove an external pile, then, it may be seized with a double tenaculum, and snipped off by means of the curved knife-edged scissors If an artery bleeds sufficiently to require a ligature, it should be tied. The following is Sir Benjamin Brodie's mode of operating for internal piles.

" I have said that internal piles are to be removed principally by ligature. You will observe I do not say they are *never* to be removed otherwise. The fact is, that when internal piles are small, it is not worth while to tie them; and they may under these circumstances be excised with perfect safety. Such a case as this will frequently occur :—A patient complains of symptoms of internal piles; he has always pain about the anus, and a discharge of mucus. You examine the parts, and find a pile, not larger than the end of your little finger, covered with the mucous membrane of the bowel, protruded, and, as it were, sticking in the orifice of the anus. You take hold of it with a double tenaculum, apply the scissars to the base, and no kind of inconvenience follows the operation. But whenever there are large internal piles, which protrude either constantly or occasionally, you ought not to venture to remove them except by ligature. In performing the operation by ligature, the first thing is to get the piles well protruded. For this purpose, you may make the patient sit over a pan of hot water, which will relax the sphincter muscle, and at the same time cause the veins of the rectum to become filled with blood. If this be not sufficient, let the patient have a pint or two of warm water thrown up as an enema; and when that comes away, the piles will probably descend with it. The piles having been by these means brought properly into view, you may let the patient lean over a table, or lie on one side in bed, with his knees drawn up, the nates being held apart by an assistant. Each separate pile must be separately tied. If the pile be of a very small size, you may just take it up with a double tenaculum, draw it out, and tie a ligature round its base. But if the piles be of a large size, you should proceed in the following manner: have a large curved needle, armed with a strong double ligature; pass the needle, carrying the ligature after it, through the base of one of the piles, and then cut off the needle. The double ligature is now divided into two single ligatures, which are tied round the base of the pile, one on one side and the other on the other side, with a single knot. Treat all the piles in this manner; and as the ligatures are applied, let your assistant draw the several threads out of your way, holding them over the nates. When each of the piles is secured in this manner (and there may be two, three, four, or five, to be thus treated), you then proceed to another step of the operation: cut off the convex portion of each pile, so as to make an opening into the cavity of the convoluted vein which forms it. Thus you take off the tension produced in the pile by the blood which it contains, and are enabled to draw the ligature tighter than before. It should be drawn as tight as possible. As the ligature is tighter, so there is less pain afterwards; so also the slough separates sooner, and the more expeditious is the cure. You have now only to complete the double knot upon each of the ligatures, and cut off the threads close to the knots, returning the piles, ligatures, and all into the rectum. It is a very simple operation; and, except when the piles are in a state of inflammation, attended with but little suffering. You are to take care, in performing it, to keep all the ligatures clear of the external parts; for if they include any of the skin, the patient suffers a great deal of pain, and much inflammation will supervene. I generally give a pretty active dose of rhubarb the day before the operation, so that the bowels may be well emptied, and that the patient may afford to go for two or three days after the operation without having an evacuation."

The ligature-threads generally separate in a week, though Sir Benjamin never takes the trouble to look after them. The subsequent management is simple. As soon as time has been given for the sores left by the separation of the ligatures to heal, small doses of the lenitive electuary should be given at night, and a lavement of cold water used every morning.

"I conceive (says Sir Benjamin) that this is not only one of the most effectual, but one of the safest operations in surgery. I should think I must have performed, or seen it performed, between 200 and 300 times. I saw one patient who died after the operation, in consequence of diffuse inflammation of the cellular membrane running up on the outside the gut as high as the mesentery; but that was a patient whose constitution was broken down by long-continued hæmorrhage, and in whom any slight accident might have produced equally bad consequences. I saw another patient, who, a week after the operation, and having been quite well in the interval, had an attack of pain in the abdomen, and shivering attended with fever, and died. I was not allowed to examine the body after death. I could not make out at the time that the symptoms had any connexion with the operation, nor do I believe that they had; but I mention the case because, as the body was not examined after death, I have no certain knowledge on the subject."

The operation then is so safe an one, that in practice all idea of risk may be properly left out of calculation.

Sir Benjamin concludes his account of hæmorrhoids by noticing a supposed case, in which the patient will not submit to an operation, or in which an operation is for other reasons inadmissible. In such a case the patient may wear what is denominated a truss for prolapsus ani, though, in point of fact, prolopsus ani is confounded both by instrument-makers and surgeons with piles. It is made with a spring which fits round the pelvis, and so far resembles a spring truss for a hernia ; but at the back part, fixed at right angles to the circular spring, there is another spring which descends behind the sacrum, taking the course of that bone, and terminating below in a pad, which rests on the anus. The elasticity of the spring supports the pad, keeps it pressed against the anus, and prevents the protrusion of the internal piles.

### Peolapsus of the Rectum.

" I have just observed," says the able lecturer, " that it is very common to confound *prolapsus of the rectum* with internal piles. This error is committed not only in common conversation, but by surgical writers ; and hence it is that no good account, so far as I know, has ever been published of the first-mentioned disease. But the difference between internal piles and real prolapsus of the rectum is this : in the protrusion of the former, the mucous membrane covering them descends, and may be seen below the anus ; but it is only the mucous membrane, there is no descent of the muscular tunics ; whereas, in the latter, the whole of the rectum comes down, and sometimes as much as twelve inches in length. I have never dissected a case of prolapsus of the rectum ; but it is impossible to examine a genuine instance of this displacement in the living person without being satisfied that the muscular tunic is protruded, as well as the mucous membrane. There being such a marked difference between prolapsus of the rectum and internal piles, nothing can be more absurd, or unscientific, than to confound these two diseases with each other."

Sir Benjamin illustrates the pathology of this disease by that of intussusceptio. They are analogous in nature ; a portion of the bowel slipping in the one instance within another portion—in prolapsus of the rectum, a portion of the bowel slipping out at the anus.

Prolapsus of the rectum occurs most frequently in children, and especially in those with large tumid bellies and costive bowels, where the whole mass of the intestine becomes too large for the cavity which contains it. Simple dissection will inform us why children are more liable to this disease than grown-up persons ; it is because the prostate gland, urethra, vesiculæ seminales, and all these parts, are not so much developed as in the adult. The attachment of the rectum to the surrounding parts does not extend so high in children as in persons of mature age, while the reflection of the peritoneum takes place lower down, and hence the rectum is more liable to be pushed out.

In adults prolapsus of the rectum sometimes occurs as a consequence of piles. The patient having been liable to the protrusion of internal piles, and the sphincter muscle having been thus continually dilated, the rectum is more liable to slip out, than it would be if this dilatation had not taken place. Sir Benjamin sees the disease in grown persons every now and then ; it has generally commenced in early life.—When prolapsus of the rectum is combined with internal piles, the latter are situated at the upper part of the prolapsus—that is, close to the orifice of the anus, forming a zone around the gut ; and the colour and appearance of the mucous membrane covering the protruded piles is altogether different from that of the membrane covering the rest of the gut.

" The inconvenience which the patient suffers from prolapsus of the rectum varies very much in different cases. Sometimes it comes down occasionally after a costive motion only, and is easily pushed up ; and when pushed up it remains in its place till some accidental circumstance brings it down again. In other cases you return it, but the moment the patient begins to walk about, down it comes again; and in instances of long standing, the bowel becomes so fixed in its unnatural position, that you cannot return it by any means, and then other inconveniences follow. The rectum having been constantly protruded, becomes inflamed from friction, ulcerated, sore, tender, painful; and where the protrusion has existed for a long time, you will find it covered by a kind of cuticle."

We extract entire the treatment of the several varieties of prolapsus, adopted by Sir B. Brodie.

" When you are called to a child labouring under prolapsus of the rectum —and these are the cases that you most frequently meet with—you will almost invariably relieve him in the following manner:—Purge him with calomel and rhubarb occasionally; be very careful about his diet, that he does not eat a great quantity of vegetable substance, which tends to fill up the cavity of the bowel, while it affords but little nourishment; and every morning let some astringent injection be thrown up. The injection which I have generally used is a drachm of tinct. ferri muriatis, in a pint of water; and two or three ounces, or more, of this, according to the age of the patient, may be injected into the rectum every morning, the child being made to retain it as long as possible. I never saw a case of prolapsus of the rectum in a child, which was not cured in this manner.

If you are consulted about an adult labouring under this disease, and it has been consequent on a protrusion of piles, the first thing to be done is to destroy the piles. Let the patient sit over a pan of hot water, and the sphincter muscle being relaxed and the parts distended with blood, the piles and rectum will all protrude together: you must then tie the piles, which you can easily do, your assistant holding the rectum on one side, while you apply the needles and ligatures on the other. Having tied the piles, you return the rectum into its proper place; and you will probably find, that in curing the piles you have also remedied the prolapsus of the bowel. But if the patient neglects himself afterwards, as the piles return so the prolapsus returns with them.

Where the disease is not complicated with piles, in those cases which occur occasionally in which prolapsus of the rectum has begun in early life, and has continued to adult age, the cure is very difficult, and perhaps impossible. The patient must be retained in the horizontal posture, for then the rectum is much less likely to protrude than when he sits up: he ought not to sit up even for an evacuation, but should have a bed-pan. Whenever the rectum protrudes, it should be pushed up again; an astringent injection should be employed daily, and the patient should be put through a course of Ward's paste. This plan affords him the best chance of a cure which he can have, but I will not say that it will always be successful. I remember trying it for a great length of time in a woman in the hospital, and, after lying many weeks in bed, when she got up the rectum came down as before; nay, it came down sometimes when she was in bed, even in the horizontal posture. In these cases, however, you may employ with advantage the truss for prolapsus of the rectum, which I mentioned as applicable chiefly to bad cases of internal piles. There was a patient in the hospital (a soldier) who had, I suppose, eight or ten inches of the rectum constantly protruded, and it could not be returned. After trying various means for a length of time, he left the hospital as bad as when he came in, and I do not know what became of him. It occurred to me afterwards, that in such a case as this it might be advisable to apply ligatures, and then cut off the protruded gut; for though the disease is not immediately dangerous, yet it must be regarded as ultimately so; and it might be worth while for the patient to run some risk at the time, for the chance of subsequent cure. I do not know that such an operation has ever been performed; but is it not deserving of consideration whether we ought not to have recourse to it in certain cases? There is a natural cure of bad cases of intro-susceptio, the analogy of which is in favour of the practice which I have just suggested. In the cases to which I allude, one portion of gut being protruded into another, the protruded portion is constricted by the edge of that into which it has passed; the circulation in it is stopped, and it sloughs away as if a ligature had been put round it. In this manner a portion of gut, eight or ten inches in length, has sometimes come away, and the patient has lived and done well afterwards. Several cases of this kind are on record; and I once had an opportunity of dissecting a patient who died when the sloughing process was taking place. If such an operation as I have proposed were to be had recourse to, the gut must be included in several ligatures, so that the orifice of it may not be obstructed, as it would be by a single one."

### Excrescences of the Rectum.

Sir Benjamin Brodie briefly mentions three kinds of excrescences which spring from the internal lining of the rectum, and may, by persons unacquainted with their nature or careless in their examinations, be mistaken for piles. The first are like uterine polypi in structure—the second is a large excrescence, not, it would appear, malignant—and the third would seem to spring from the combination of external piles and filth.

1. "Here," says Sir Benjamin, "is an excrescence—a sort of polypus. It is, as you see, of a small size, but I have seen them as large as the finger. It seems to be of the same structure as the polypus of the uterus. This kind of excrescence is by no means uncommon. Sometimes there is a single one; at other times there are two or three growing from the mucous membrane. In some instances they occasion the patient scarcely any inconvenience, while in others they give rise to the most extraordinary suffering. What is is it that makes this difference? The patient suffers in those cases in which the excrescence comes down when the bowels act, and gets pinched by the sphincter muscles. Under these circumstances it is liable to become ulcerated, and then the pressure of the sphincter ani always induces excessive pain, which continues not only till the excrescence recedes, but for some time afterwards. A lady sent to me, complaining of what she called very bad piles. On examining the rectum, I discovered a little polypous excrescence, in a state of ulceration, sticking in the sphincter muscle. I took hold of it with a pair of forceps, and snipped it off with the scissors. She felt hardly any inconvenience from the operation, but, to her surprise, though she had been enduring a great deal of pain, and had been miserable for months, from this moment she was well. A lady, not long since, came to my house, from a distance in the country, in whom most severe sufferings were occasioned by one of these polypi being ulcerated and entangled in the sphincter muscle. I immediately snipped it off; she was completely relieved; went home, I believe, on the same day, and I have no doubt has been quite well ever since."

2. The second species of excrescence described by the able lecturer, is large as we have said, and apparently not of a malignant nature. He describes it by a preparation which we cannot shew, and by a case which we can.

"This preparation," he tells his pupils, "I removed from an old lady, 80 years of age. She sent to me, complaining of pain about the rectum, and hæmorrhage. I thought there were probably internal piles, and that it was not worth her while, at so advanced an age, to go through any operation, and I prescribed her some trifling medicine. She sent to me again, to say that she had lost a great deal of blood, and could not pass an evacuation from the rectum without the greatest difficulty. I introduced my finger and found a large excrescence, of which this specimen is only a portion. It seemed to be a matter of necessity that something should be done for the patient's relief: I therefore introduced my fingers into the rectum, gradually dilated the sphincter muscle, took hold of the excrescence, pulled it down, tied a ligature round its neck, and then snipped it off below the ligature. No harm followed the operation; the patient was perfectly relieved, and lived some two or three years afterwards. I believe the excrescence returned before death, but still she suffered no inconvenience from it."

3. The third kind of excrescence would seem to resemble condyloma in its origin—irritation of the cutis from moisture and filth.

"These excrescences were, I believe, originally external piles, and they are not very uncommon. I mentioned in the last lecture, that when the cavities of external piles become obliterated, they generally form flaps of skin, which gradually waste; but sometimes diseased action takes place in them, and they become converted into excrescences similar to those which grow from the nymphæ of women. They are generally connected with dirty habits: the parts get irritated by the dirt, and so the piles become converted into these excrescences, into which they would not be converted in a more cleanly person."

We beg to direct attention to these valuable observations. They bear the common stamp of all the writings of the able and estimable Baronet—simplicity and truth. The reader on this, as on other occasions, is struck with the plain unvarnished sense that marks the acute observation and induction of a philosophical mind. There is none of that erring spirit of speculation from which so few medical writers are free, but plain truths are told in an unaffected style. This it is which gives to all that falls from the mouth or pen of Sir Benjamin Brodie, the value which pupils and the profession alike combine to attach to it. The class of St. George's Hospital enjoy the advantage of listening to the precepts and observing the practice, of this, we say it in absolute sincerity, the most philosophical (if philosophy be the pursuit of truth)—the most philosophical, we repeat, of modern surgeons.

---

### SOME REMARKS ON ULCERATIONS OF THE RECTUM OCCURRING IN CONNECTION WITH VENEREAL SECONDARY SYMPTOMS.

By HENRY JAMES JOHNSON, late House Surgeon to the Lock Hospital.

I am not aware of the existence of any good account, or indeed of any account at all, of the ulcers of the rectum I am about to describe. In this, as in many other instances, writers on venereal diseases would seem to have contented themselves with copying or criticising each other's observations, without referring to, or, at all events, with only partially studying the facts that Nature presents. Some speculative points have engaged their attention, and excited amusing or acrimonious discussion. The origin or the introduction of syphilis, a question which can never be decided at all, or if decided, would lead to no useful result—the true chancre, a something like the Basilisk in Zadig, which has never yet been found, and if found would be valueless—the abstract identity of syphilis and gonorrhœa, two diseases which display the most opposite phenomena, and require the most opposite methods of treatment—such matters as these we find liberally mooted, and writer after writer contributes his small share to their elucidation or confusion. But a careful observation of the malady as it is—a rigorous study of its forms—and a plain and straight-forward analysis of its varieties—these practical excellencies unhappily are not evinced by the cloud of authors, the mere enumeration of whose names and works forms a bulky volume.*

It would be tedious to point out the many venereal symptoms which have received an inadequate degree of attention—inadequate, at least, to the practical result of distinguishing their

* This is a fact. Some gentleman shewed me a catalogue of this description. He had I believe a large proportion of the works. I cannot suppose him guilty of reading them.

varieties and discriminating their treatment. It is true that numerous forms of venereal sores have been described by authors. Yet in these descriptions we observe the utmost discrepancy and vagueness, and not only do no two writers employ the same names, but they do not present the same images of the same things. There are many varieties of venereal ulceration of the throat—many of venereal eruptions on the skin; but I know of no work in which they are plainly and faithfully portrayed, none which have corresponded with the facts that I have witnessed. Nature then must vary and contradict herself, or observers must be careless and inaccurate. The choice of these alternatives may be safely left to the judgment of the reader.

Without pursuing farther at present this train of general remark, I shall throw together a few desultory observations on some instances of venereal ulceration of the rectum, that have fallen under my observation in the last two or three years. Those instances have not been numerous, with all the opportunities that the Lock Hospital presents for extensive observation. Ulcerations of the rectum then, primary or secondary, are probably not frequent, though certainly they are more common than the general silence on the subject would imply. The following brief remarks may be considered the condensed expression of the particulars of the cases I have seen.

### I. Ulceration of the Rectum in connection with Condyloma.

I am not aware of having seen distinct primary ulceration except in connexion with condyloma. Condylomata not unfrequently ulcerate, and the ulcerated fissures may reach the anus or even pass for a slight distance within it. The presence of the condylomata is sufficiently distinctive of this species of ulcer.

Ulcerated condylomata, or condylomatous ulceration in the vicinity of the rectum, is frequent in the lower class of prostitutes. The discharge from the vagina dribbling down and collecting in the perineum and between the nates, gives rise to condylomata; uncleanliness, irritation, and perhaps the nature of the disease itself, make those condylomata ulcerate. The disease is thus produced in the filthy women that infest the streets, and form a large proportion of the inmates of the Lock Hospital. But it also occurs under circumstances where its presence would not be so natural. The following case, which is curious in more respects than one, bears upon the present subject.

*Case.* A gentleman who had been a patient of mine previously with secondary ulceration of the throat, and an eruption consisting of copper-coloured stains, came to me lately with what he said was an excoriation. He had had connexion on the preceding night with a female whom he said he could not suspect, a *lady* who was kept by a friend of his own. The excoriation was seated on the inner prepuce, at its angle of reflection behind the corona glandis. It was extensive, superficial, with a slightly yellowish surface. I told the gentleman that the sore had something more than the aspect of a simple abrasion of surface. He was confident that he was right, and simple means were adopted and pursued for a fortnight. At the expiration of that period the sore had more decidedly the character of syphilis. I insisted on a change of treatment. He wished the lady to be submitted to an examination. To this she readily consented, declaring at the very instant that her clothes were raised, that she was free from all complaint. A very slight inspection disclosed a condylomatous sore at the inferior commissure of the vagina, and another at the anus, partially extending within that aperture. This settled the question at once. I immediately put the gentleman on a course of mercury, and the sore upon the prepuce, that had previously been spreading under simple treatment, cicatrized with remarkable rapidity.

The chief interest of this case is connected with the communication of condylomatous ulceration. The condyloma, it is well known, or at all events it is believed, may result from the irritation of gonorrhœal matter. The condyloma so produced very frequently ulcerates, and forms a sore. That sore was communicable in the case I have related. Of condylomatous sores I have seen very numerous examples, but I never before had an opportunity of observing the ulcer that they were capable of giving rise to. That ulcer has, or in the case in question had, the characters of distinct syphilitic ulceration—a yellow surface—a thin edge rather undermined—and a distinct substratum of induration.*

I will take the liberty of introducing here a few observations appended to a former paper upon condyloma. They bear upon the present case though not upon the present subject.

"It will probably have occurred to the minds of some of the readers of this paper, that a striking singularity attending condyloma is its origin in gonorrhœal discharge, and its power of giving rise to secondary symptoms. We not unfrequently hear surgeons talk of gonorrhœal sores and gonorrhœal secondary symptoms. So far as my observation has extended, their ideas have been as vague as their language is indefinite. Unless condylomatous ulcerations be considered gonorrhœal, I have seen none, nor have I ever witnessed a fair example of secondary symptoms following uncomplicated gonorrhœa. Making no pretence to theoretical speculations, I merely offer the results of observation. If that observation be correct, condyloma would appear to form some intermediate state between gonorrhœa and syphilis."

Condylomatous ulceration of the rectum sometimes presents itself in men. As in them there is no surface like that of the vagina to supply a free discharge, which flows towards the anus and collects in its vicinity, we must look for some other efficient cause. Charity may suppose that filth is adequate to the production of the effect. But the surgeon of a hospital devoted to venereal cases is compelled to think, indeed must feel convinced, that in some instances an agency more criminal than filth has given rise to the disgusting complaint. During the period of my residence in the Lock, I had under my care as out-patients two men, with extensive condylomatous ulceration of the rectum, in whose cases there was little reason to doubt that the cause was such as I have only dared to hint at.*

I do not think it necessary to say any thing here on the treatment of condylomatous sores. For that I must refer to the paper already quoted, and contained in the 41st number of this Journal.

### II. PHAGEDÆNIC ULCERATION OF THE RECTUM.

The ulcers of the rectum which I have witnessed in connexion with secondary symptoms have been of three descriptions. Of the two first I have seen severally but a single case; of the last I have observed many.

*Case.* A woman was received in the Lock Hospital under these circumstances. She appeared to be in an indifferent state of health. She had not been on the town, but had merely had intercourse with two or three persons. About three months previously to her admission, she had first found a sore and discharge from the vagina. For this she spontaneously took mercury so as to affect her mouth. She got better,

---

* I have freely used the terms condyloma, and condylomatous sore. For the sense in which these terms are employed, the reader must recur to the 41st number of this Journal. If he has not read the paper contained in it, he will not understand the present case. The desire of avoiding repetition is my excuse for contenting myself simply with the present reference.

* A slight acquaintance with continental medical literature is sufficient to establish the melancholy fact—that primary ulcerations of the rectum are frequently abroad in both sexes. Those ulcerations probably display their share of the varieties observed in primary sores upon the genitals.

but after a short time relapsed, and now began to suffer from pain in the rectum, and, as she thought, from piles. She again put herself under a course of mercury which she carried to profuse salivation. This reduced her greatly, and had occasioned the indifferent condition of health remarked on her reception in the hospital.

On examination there was discovered at the junction of the nympha and labium of the right side, a deep, sloughy-looking sore, with thin edges, a foul yellowish red surface, and little or no surrounding induration. The sore bled readily. A bloody offensive discharge also issued from the anus. There was no ulceration of the rectum within sight, but on introducing the finger into the gut, an oval ulcer, larger than a half-crown-piece was distinctly felt on the posterior wall of the bowel. So far as the discharge went, and as well as the finger could determine, the characters of that ulcer resembled those of the sore in the vagina. On the left hip near the great trochanter was a large scab of rupia, and one or two other but smaller rupia spots appeared on the lower extremities.

Was this a primary or secondary ulcer of the rectum? The symptoms that seemed to mark its commencement and the rupia had appeared nearly simultaneously, some time after the establishment of the sore in the vagina. Whether primary or secondary, the nature of the sores—the rupia—the bad health —and the course of mercury so lately taken were ample reasons for objecting to give more. The patient was therefore ordered sarsaparilla, at first in the form of decoction and afterwards in that of syrup. Good diet and wine were conjoined with the administration of the sarsaparilla. Various applications were used to the ulceration in the rectum, but a moderately-strong solution of the nitrate of silver, injected into the rectum with a syringe, was the application that agreed the best and was employed the most.

At first, the patient gradually grew worse. The sore in the vagina increased in size, though not materially —the rupia-scabs separated, and left ulcerations of a circular form, and disposed to spread with a yellow border and undermined edge; that near the trochanter attained the dimensions of the central part of a cheese-plate—the ulcer in the rectum manifestly extended, for the bloody discharge was profuse, and the pain, at all times great, became excruciating on the passage of a motion —and, finally, the patient became extremely emaciated, and so reduced in strength that she seemed not unlikely to sink.

Under these circumstances, small quantities of mercury were tried in conjunction with the sarsaparilla and other tonics. But she did not amend, on the contrary, she deteriorated under this treatment. The following plan was then adopted and steadily pursued.—Full doses of the syrup of sarsaparilla were given for three weeks or a month at a time, discontinued for a week, and then repeated as before. Suppositories of opium and belladonna were introduced, in order to relieve the excessive pain—occasional mild doses of rhubarb, combined with hyosciamus and laudanum, regulated the condition of the bowels—injections of the solution of the nitrate of silver were made use of. In addition to these medicines the patient was allowed a generous diet, and half a pint of port wine daily.

So soon as this plan had been for a short time in steady operation, a perceptible amendment ensued. The large and sloughy rupia sores slowly healed —the vaginal sore healed also—and the discharge and other symptoms of the ulceration in the rectum tardily subsided and finally disappeared. Yet ten months were consumed in effecting this, and at the expiration of that long period when the patient left the hospital for the country, she was so reduced in strength, that she was scarcely able to walk down the hospital stairs. Prior to the adoption of the method of treatment detailed above, she appeared to have little chance of ultimate recovery.

It is perhaps uncertain whether the ulceration of the rectum was primary or secondary in its character. The probabilities are in favour of the latter supposition, for the symptoms denoting its existence appeared simultaneously with rupia. Whether primary or secondary, its connexion with the rupia, the condition of the health, the danger of the patient, and, I may add, its own appreciable characters, are sufficiently decisive of its phagedænic nature—the term phagedæna implying no more than a foul condition of the sore, and a disposition to extend by a combination of sloughing and of ulceration, without involving any hypothetical considerations whatever.

The plan of treatment which answered in this instance was, repeated short courses of the syrup of sarsaparilla, with brief intervals between each course. I may take the opportunity of remarking that in cachectic cases, where the health is impaired—and the ulcerations whether primary upon the genitals, or secondary in the throat or on the skin, assume the phagedænic character—and, finally, in cases of affection of the periosteum or bones, too frequently combined with, but occasionally independent of the form of ulceration I have mentioned—in such cases, where sarsaparilla and tonics must be long continued, the most useful mode of exhibiting the former is in short courses of this description. Between each course some mild aperients should be given. The infusion of rhubarb with infusion of orange-peel and a few grains of carbonate of soda answer well. Sarsaparilla sometimes occasions relaxation of the bowels, but it usually disposes to constipation, and purgatives are commonly required during its continuance.

### III. Ulceration of the Rectum, with Ulceration of other Mucous Membranes.

If the nature of the preceding case is dubious, I mean if it is uncertain whether the ulceration was primary or secondary, the same cannot be said of the one I shall now describe.

*Case.* A young man, the proprietor of a public-house in the Strand, applied to me a few months ago, on account of a peculiar ulceration of the lip. Nearly the whole of the exposed part of the lower lip was occupied by an irregular superficial ulceration. It was situated on the border, and partly on the internal surface, where the lip is invested by the mucous membrane,* but it did not spread beyond the red part on the external skin. It was so superficial, that it looked like the ulceration left by those burns which only destroy the superficies of the cutis. It was white in its colour, and its irregularity was partly occasioned by its healing in one direction and spreading in another. The spreading edge was always an abrupt one, and peculiarly white. On examining the throat, I discovered precisely similar ulceration on the soft palate and on either tonsil. There was no eruption on the skin.

It appeared that, two or three months previously, the patient had suffered under what he considered, and a surgeon treated, as ordinary gonorrhœa. The ulceration of the lip had existed for about a week; he was not aware of the presence of any ulceration in the throat. He denied having had any venereal sore.

Never having witnessed secondary symptoms after gonorrhœa, I thought that this was a proof of their occurrence, and an example of their character. An inspection of the scrotum staggered me in this opinion; in fact, it appeared to shew that it was erroneous. On the inferior surface of the penis, and anterior surface of the scrotum, were several almost tubercular deposites in the cutis, cupped in their centre, where some were slightly ulcerated still, and some presented the cicatrices of ulceration. These sores were such as I have frequently seen, and which have always displayed the phenomena and history of syphilitic ulcers. In order to observe the progress of the symptoms, I ordered some bread pills and some mild aperients.

The ulceration of the lip slowly travelled over its entire red border, and extended downwards on its inner surface. The ulceration of the soft palate also

* I use this term to make myself understood. The inner surface of the lip is covered with cuticle, beneath which is seated what geologists would denominate a *transition* structure between cutis and mucous membrane. But the anatomists prefer the latter.

spread to the contiguous cheek and gum; but the characters of neither essentially varied, and it still remained as superficial as before, and still retained the peculiar whiteness. Other symptoms, however, were developed. Ulceration of a similar description was established on the tarsal margin of the palpebral conjunctiva of the left eye; it began at the inner angle, close to the punctum lacrymale, and gradually spread outwards. Soon after this, the other eye became affected in exactly the same manner. And, finally, ulceration, still of the same character, white and superficial, appeared upon the margin of the anus, or rather just within it, not extending on the common cutis. These ulcers of the lower portion of the rectum were productive of some, but not great pain.

I now put the patient on a course of three grains of blue-pill twice daily, and combined sarsaparilla with the mercury. Black wash only, the lotion previously employed, was applied to the ulcerations of the rectum and the lip.

Under this treatment all the ulcers healed, the deposites in the cutis of the scrotum were removed, and perfect health was re-established. I have seen the patient lately, and no return of the symptoms has occurred.

White ulceration of the tonsils is the most frequent secondary symptom after ulcerated condyloma. In one case, I saw also general, white superficial ulceration of the lower lip, exactly resembling the ulceration of the lip in the preceding case. In that case, one remarkable feature is the co-existence of a peculiar ulceration in the mucous membrane of the lip and throat, the mucous membrane of the eye, and the mucous membrane of the rectum. I have seen no other case precisely similar to this.

### IV. Ulcers of the Rectum, accompanying various Secondary Symptoms.

This form of ulceration of the rectum is certainly not very rare. I have seen five or six cases of the kind. It is usually observed in women; at least I have seen it but once in the male.

The patient's attention is usually first directed to the part, in consequence of extreme pain on attempting to discharge the fæces. He frequently observes a small quantity of blood in the motion, and one or other, or more frequently both these circumstances, induce him to consult the surgeon.

The state of the rectum varies with the stage of the complaint, for at first there is usually one ulcer only; but several are formed in succession, and some may run into each other and become confluent. More frequently, one forms upon one side of the gut, and others are developed consecutively round it. The ulcers that form first are on the margin of the anus, where the skin and the mucous membrane are continuous—those which appear afterwards are higher in the gut, and the late ones are out of sight (unless the speculum be used), and require examination with the finger. To detect these ulcers, the anus must be stretched open, and its folds undone. If this is neglected, the small sores will sometimes escape observation. Sometimes these ulcers, situated round the anus, assume a radiated form.

The character of the ulcers is well marked. They commence as a yellow spot, evidently produced by an ulcerated aperture in the cuticle, displaying a small yellow ulcer in the cutis. The ulceration enlarges, but seldom attains the dimensions of a sixpence. Its surface is yellow, at first slightly cupped—afterwards the seat of small spongy granulations. The edge is undermined. When cicatrization commences, the yellowness is, of course, exchanged for vascularity, and when cicatrization is completed, little perceptible induration remains.

These ulcers occasion excessive pain. This is violent on attempting to void a stool, and remains for hours afterwards. The patient dreads the idea of a motion. But pain comes on in paroxysms, independently of the action of the bowels, and sometimes it is so severe at nights as to keep the patient awake in agony. Examination of the bowel by the surgeon generally causes a great deal of suffering; so does the application of the necessary remedies.

I am not aware of the natural course and termination of these ulcers, independently of the influence of medicine. The pain and distress which they always create drive the surgeon to decisive treatment, and prevent the performance of the rational experiment of non-interference.

I observed that I had witnessed five or six examples of this description of ulceration of the rectum. In all, secondary symptoms were present; yet it is a source of considerable regret, that I am enabled to speak positively to the nature of those secondary symptoms in but three.

1. In a girl at the Lock Hospital, who was affected with these ulcers, there was a yellowish, superficial ulcer at the inferior commissure of the vagina, some spots of syphilitic psoriasis guttata on the trunk and lower limbs, and superficial yellowish ulceration of the tonsil. This patient was suddenly dismissed for improper conduct, and I am not acquainted with the termination of the case.

2. Another girl was received into the Lock Hospital, on account of suppurating bubo. There was copious discharge from the vagina, and small superficial ulceration on the outer surface of the right nympha. Soon after her admission, she became affected with superficial ulceration in the throat, and after that she was attacked with violent pain at stool, and a slight discharge of blood with the motion. On examination, I discovered two or three ulcerations in the rectum, situated near the outer margin of the anus. I ordered the patient calomel and opium—the application of the nitrate of silver every other morning to the ulcers—and suppositories of opium and belladonna at night. She perfectly recovered.

3. The following case occurred to me lately. A young gentleman, in a very indifferent state of health, applied to me on account of ulceration of the throat, and some other symptoms of a venereal character. The ulceration was situated on the tonsils—it was yellowish, superficial, attended with some enlargement of the tonsils. On the body and limbs there were a few copper-coloured stains, attended with distinct, though not with great deposition in the cutis; the surface of these stains was rather inclined to desquamate than to scab. There was chronic inflammation of one testis, with fluid in the upper part of the tunica vaginalis. This patient was not aware of any distinct primary sore. He had been under some very good surgeons for the ulcers in the throat and the eruption, and had been told that they were not venereal, and that he need not take mercury. Yet sarsaparilla, and the other items of the treatment which they recommended, had proved inadequate to effect a cure. I felt convinced that the ulceration of the throat was syphilitic, and stated my opinion to the patient. But his health appeared unequal to support the exhibition of mercury, and sarsaparilla and country air were the measures recommended.

He went into the country, but speedily returned with what he thought were hæmorrhoids. He had violent pain on going to the stool, and a slight discharge of blood from the rectum. On examination, I found that an ulcer, of the character that has been sufficiently described already, was seated at the margin of the anus, on the right side. Soon after this, another ulcer formed on the left side of the gut; and next, a third, upon its posterior surface. These ulcers were all external, that is, they could be seen on moderately opening the aperture of the bowel. They successively healed under the treatment adopted; but now another sore formed within the gut, and could be felt with the finger on its posterior wall; it appeared to be about the magnitude of a shilling. This also cicatrized and no return of the ulcerations of the rectum has been noticed.

From the time when the ulcers of the rectum first appeared till their perfect cicatrization, a period of six or seven weeks, this gentleman suffered occasionally to a great degree. The ulcers, at their commencement, were severally productive of the greatest agony, especially at stool. Each ulcer existed from ten days to a fortnight, and, when one was healing, another was in the stages of formation and increase.

The treatment which I followed up was this. Two grains of calomel and half a grain of opium twice daily for two months—sarsaparilla—nutritious diet, least calculated to make fæces—castor-oil in small quantities every other morning—and latterly, with almost immediate relief, a suppository of belladonna and opium. The ulcers were touched every third day with the nitrate of silver in substance, and an injection of the solution of the sulphate of copper was thrown into the rectum night and morning. When the ulcer which has been mentioned formed internally, it was found impossible to touch it properly with the caustic in the solid form, and an injection of its solution, twenty grains to the ounce, was introduced by means of a silver syringe, every third day, in its place.

The treatment of these distressing ulcers of the rectum, distressing, from the pain which they occasion, should, so far as I know, be as follows—at all events I have found this answer the best. Calomel and opium, so as gently to affect the mouth—the judicious employment of sarsaparilla and tonics—small doses of castor-oil, to obviate the occurrence of bulky fæces, and diet selected and adapted for the same purpose—rest in the horizontal posture; such are the general principles of treatment which I have found most applicable to the disease. The local treatment is of paramount importance. The sores should be touched, every second or third day, with the lunar caustic, and, in the interim, injections of zinc or copper, or some other stimulant or gentle escharotic, should be used. When the ulcers are not perfectly accessible to the solid caustic, its strong solution should be employed as an injection. The bidet, or the hip-bath, should be used two or three times daily. When the pain is severe, particularly at nights, an opiate suppository is almost always useful.

Such are the remarks I have to offer on the subject of venereal ulcerations of the rectum. They are imperfect, it is true; but, so far as they go, they are exclusively the result of observation, and their very imperfections may stimulate others to endeavour to supply the deficiencies which they exhibit.

---

### MISCELLANIES.

Birmingham School of Medicine.

